# scBalFlow: a staged flow matching framework for imbalanced single-cell drug perturbation prediction

**DOI:** 10.1093/bioinformatics/btag682

**Published:** 2026-09-15

**Authors:** Hanwen Lyu, Jiawei Luo

**Affiliations:** College of Computer Science and Electronic Engineering, Hunan University, Hunan 410082, China; College of Computer Science and Electronic Engineering, Hunan University, Hunan 410082, China

## Abstract

**Motivation:**

Conventional drug perturbation prediction models typically employ end-to-end encoder-decoder architectures, directly mapping control samples and perturbation conditions to post-perturbation gene expression profiles. However, these approaches widely overlook the severe class imbalance inherent in perturbation datasets, leading to a predictive bias toward weakly responsive samples.

**Results:**

To address this bottleneck, we propose scBalFlow, a decoupled two-stage training framework. The first stage predicts the perturbation response intensity under given conditions, employing a Gaussian-Augmented Inference (GAI) strategy to counteract data imbalance. Crucially, the second stage bypasses weakly responsive conditions, while utilizing a Flow Matching model to synthesize highly responsive samples. Comprehensive evaluations on large-scale benchmarks, including SciPlex3 and McFarland, demonstrate that scBalFlow effectively overcomes the imbalance issue and significantly outperforms existing state-of-the-art methods on imbalanced datasets, particularly in capturing complex distribution shifts and maintaining single-cell distributional consistency.

**Availability and implementation:**

The source code and datasets are available at GitHub https://github.com/hanwenlv-cmd/scBalFlow and Figshare with doi:10.6084/m9.figshare.33137447. The datasets of SciPlex3, ComboSciPlex, and McFarland underlying this study are available via the pertpy package. Alternatively, they can be downloaded manually from https://exampledata.scverse.org/pertpy/srivatsan_2020_sciplex3.h5ad for SciPlex3, https://exampledata.scverse.org/pertpy/combosciplex.h5ad for combosciplex, and https://exampledata.scverse.org/pertpy/mcfarland_2020.h5ad for McFarland.

## 1 Introduction

High-throughput drug screening facilitates the identification of potential therapeutic candidates and genetic targets by analyzing cellular gene expression profiles across diverse combinatorial perturbation conditions (Carnero 2006). However, the vast combinatorial space of these conditions makes exhaustive experimental exploration prohibitively expensive and time-consuming (Menden *et al.* 2019). Consequently, deep learning approaches have emerged to predict the effects of unseen perturbations by training on existing datasets (Aliper *et al.* 2016).

Current methods typically employ encoder-decoder architectures, such as Conditional Variational Autoencoders [CVAEs; Kingma and Welling (2014] or Conditional Autoencoders [CAEs; Lopez *et al.* (2018)], which utilize control profiles as inputs and perturbation conditions as covariates to predict post-perturbation states. Despite achieving reasonable accuracy in predicting mean gene expression levels, these models often fail to capture the detailed distribution of individual samples. To overcome this issue, generative models based on multi-step iterative refinement, such as diffusion models (Ho *et al.* 2020) and conditional flow matching (Lipman *et al.* 2023), have gained significant traction. However, these methods sharply increase training and inference costs. More crucially, they share a fundamental vulnerability: they are highly prone to overfitting weakly responsive gene expression profiles.

This vulnerability stems from a core characteristic of drug perturbation data: class imbalance, where only a small fraction of candidate compounds triggers significant biological responses. To characterize the imbalance in drug perturbation responses, we visualize the similarity between perturbed and control expression profiles across different doses in Fig. 1. The distributions show that most perturbation conditions remain highly similar to their corresponding controls, revealing a strong prevalence of weakly responsive conditions. Because previous approaches predominantly rely on end-to-end training paradigms, the models are implicitly forced to map a weakly responsive sample to another nearly identical one. This leads to model degradation, where the network simply converges to predicting the global mean expression of the control group. While standard imbalance-mitigation techniques (e.g. resampling or weighted loss functions) can partially alleviate this, end-to-end models inherently require massive parameter capacities to reconstruct high-dimensional gene expression profiles. Consequently, they remain highly susceptible to overfitting despite these corrections.

**Figure 1:**
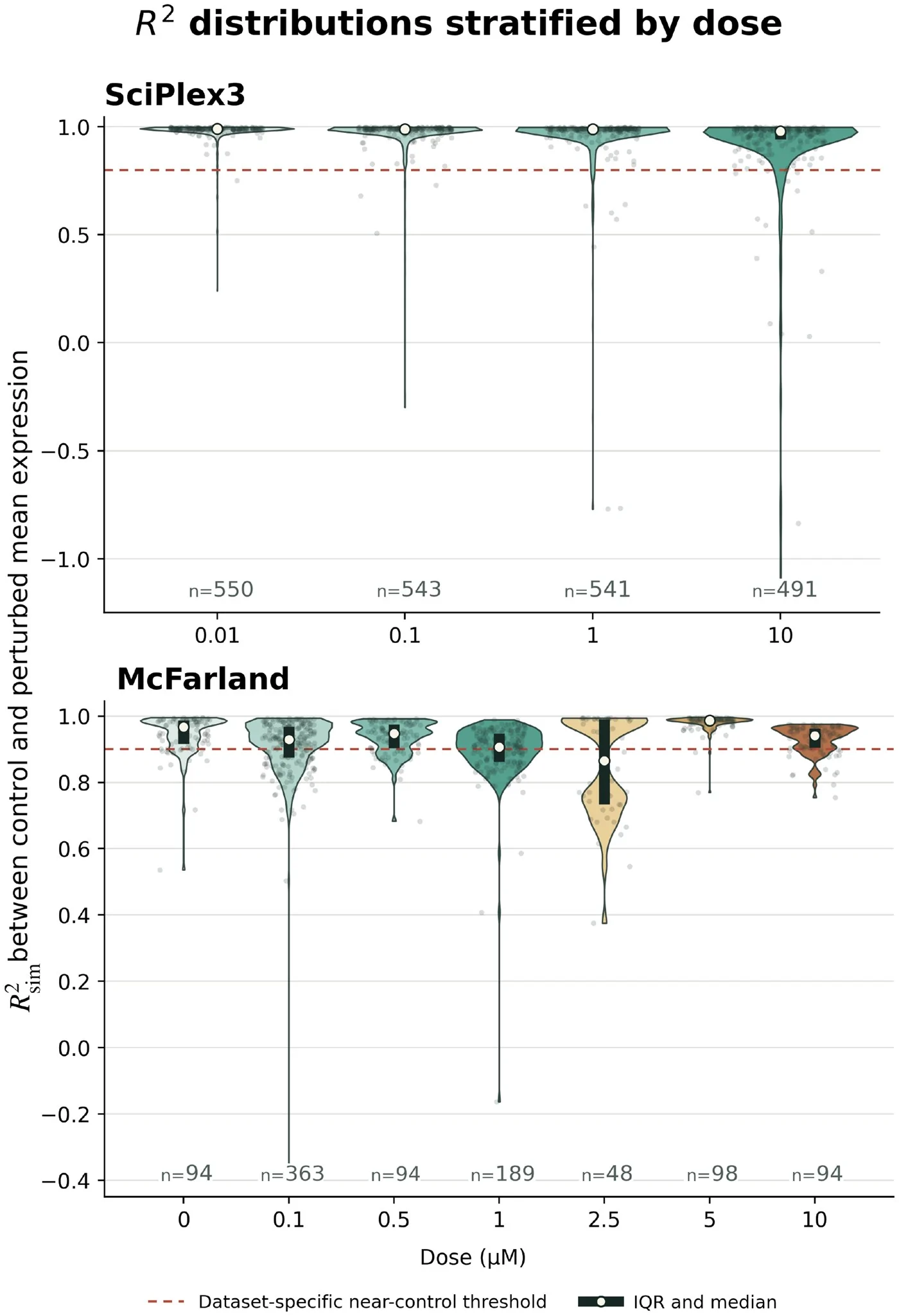
Dataset imbalance. This figure visualizes the distribution of control–perturbation similarity across different doses in SciPlex3 and McFarland. $R_{\text{sim}}^{2}$ close to 1 indicates that the perturbed profile remains globally similar to the control, whereas a lower value indicates a stronger global transcriptomic departure. The correlation between $R_{\text{sim}}^{2}$ and biological response intensity is shown in Fig. 1, available as supplementary data at *Bioinformatics* online. Dashed thresholds: SciPlex3 $R_{\text{sim}}^{2}$ = 0.8; McFarland $R_{\text{sim}}^{2}$ = 0.9. Notably, dose alone does not explain the imbalance: although higher doses in SciPlex3 slightly increase the fraction of responsive conditions, many perturbations remain near-control. McFarland does not permit a clean within-drug dose-effect analysis because most drugs were measured at only one nominal dose, making dose and drug identity inseparable.

To address these challenges, we propose scBalFlow, a staged training framework that decouples the prediction task into response identification and expression synthesis. The framework consists of two primary stages: Response Prediction and Gene Expression Prediction. In the first stage, we quantify the cellular response level by calculating the $R_{\text{sim}}^{2}$ score between the control and post-perturbation gene expression profiles. Conditions less than a predefined $R_{\text{sim}}^{2}$ threshold are classified as “highly responsive” (the minority class). Simultaneously, we implement Gaussian-Augmented Inference Loss [GAILoss; Ren *et al.* (2022)], a specialized weighted loss function designed to handle severe class imbalance. The second stage employs a self-attention-based Flow Matching generative model. Guided by perturbation conditions to control the generation trajectory, the model learns to transform standard noise into the target perturbed gene expression distributions. Since the post-perturbation distribution for weakly responsive conditions is virtually identical to the already-known control group, our staged framework effectively eliminates this unnecessary computation. Consequently, the generative model can dedicate its entire representational power exclusively to modeling the complex distribution shifts of genuinely responsive samples, thereby improving both generation quality and computational efficiency.

### 1.1 Related works

Traditional methods primarily rely on end-to-end training paradigms, typically employing lightweight encoder–decoder architectures. For instance, PRnet (Qi *et al.* 2024) utilizes a CAE framework, while scGen (Lotfollahi *et al.* 2019) and biolord (Piran *et al.* 2024) leverage CVAEs to learn latent representations of cellular states, achieving perturbation synthesis via linear vector arithmetic in the latent space. Similarly, CPA (Lotfollahi *et al.* 2023) and chemCPA (Hetzel *et al.* 2022) employ Adversarial Autoencoders to disentangle perturbation covariates, enabling counterfactual prediction.

Recently, generative models based on Flow Matching, such as CellFlow (Klein *et al.* 2025) and scDFM (Yu *et al.* 2026), have been introduced to achieve superior distributional alignment. These methods estimate a continuous vector field that transports samples between source and target distributions, improving the distributional alignment between predicted and observed perturbed cell populations. Despite their modeling differences, these approaches are commonly trained across the full set of perturbation conditions. In contrast, scBalFlow adopts a staged strategy that first identifies perturbation conditions requiring generative modeling and subsequently applies flow matching only to these selected conditions.

## 2 Materials and methods

The overall architecture of **scBalFlow** is illustrated in Fig. 2. The framework consists of two main stages: (i) **Response Prediction** in the first stage, which utilizes a Residual Multi-Layer Perceptron (ResMLP) to predict the $R_{\text{sim}}^{2}$ score, using a concatenated vector consisting of the encodings of the drug, cell type, and dosage as its input. (ii) **Expression Prediction** in the second stage, which employs a Flow Matching generative model constructed with multi-layer self-attention blocks. This stage uses Gaussian noise as the latent space and perturbation conditions as control signals via Adaptive Layer Normalization [AdaLN; Peebles and Xie (2023)] to iteratively generate perturbed gene expression profiles through ODE integration.

**Figure 2:**
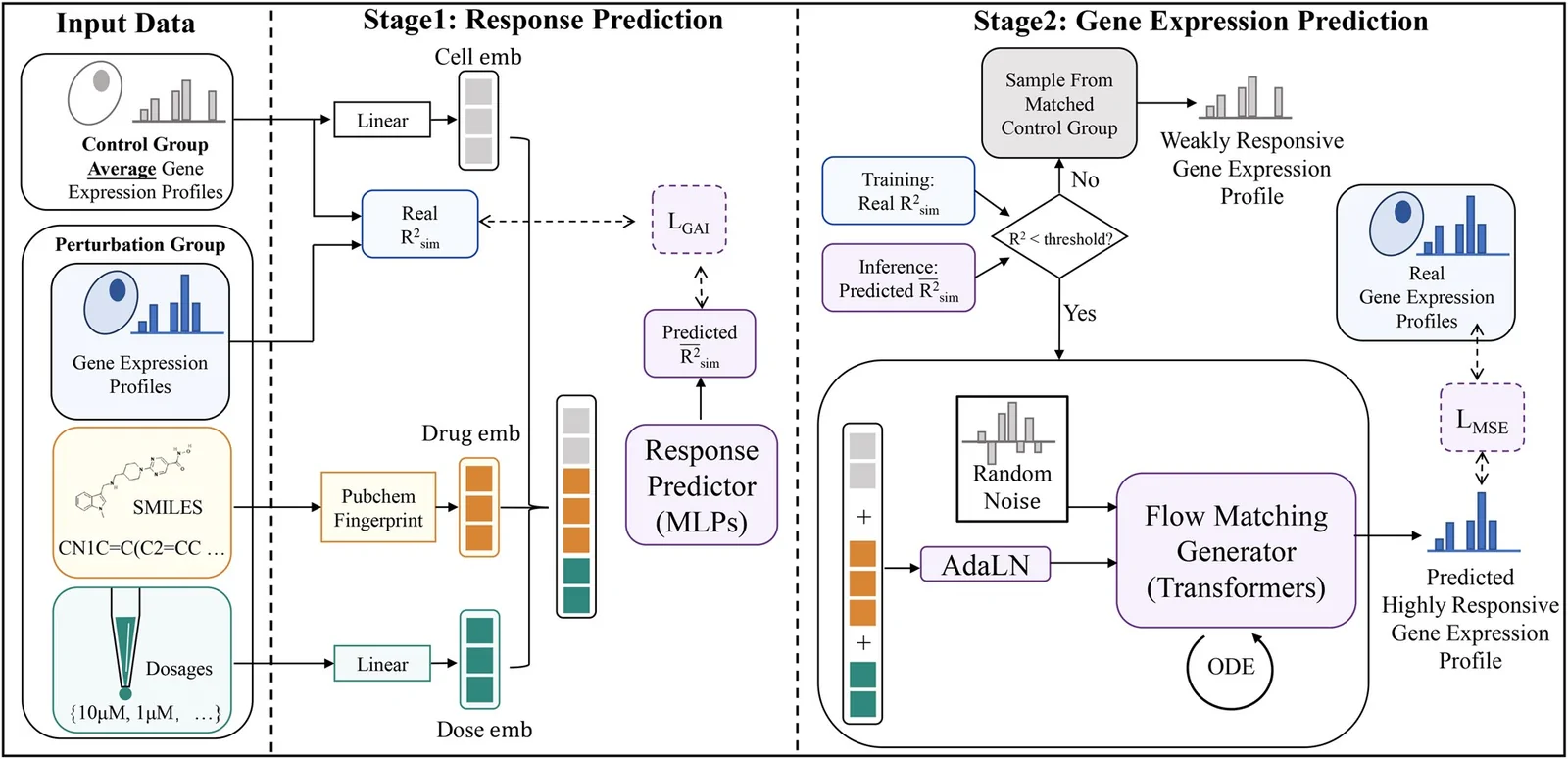
The overall architecture of scBalFlow framework. scBalFlow adopts a two-stage strategy to address the imbalance between weak and strong perturbation responses. The data are divided into matched control and perturbation groups. Cell-type representations are obtained by linearly projecting the mean expression profile of the corresponding control cells, drugs are represented using PubChem fingerprints, and dosages are encoded through a linear layer. In Stage 1, the precomputed coefficient of determination between the mean control and perturbed expression profiles, denoted as $R_{\text{sim}}^{2}$, serves as the response-similarity label. The three embeddings are concatenated and fed into an MLP-based response predictor, which estimates $R_{\text{sim}}^{2}$ and is optimized using GAI Loss. In Stage 2, the real $R_{\text{sim}}^{2}$ values are used during training to route highly responsive conditions to the flow-matching generator, whereas weakly responsive expression profiles are generated by sampling from the matched control group. During inference, the predicted $R_{\text{sim}}^{2}$ from Stage 1 is used for routing because post-perturbation expression profiles are unavailable for unseen conditions. The two stages use the same encoding schemes but do not share encoder parameters.

### 2.1 Response prediction stage

#### 2.1.1 Training data

In this stage, the model does not directly process high-dimensional gene expression data. Instead, it takes the perturbation condition cond as input, which is composed of the drug molecule (SMILES), the target cell type (cell), and the drug dosage (dose). Given $N_{\text{chem}}$ drugs, $N_{\text{cell}}$ cell types, and $N_{\text{dose}}$ dosages, the total number of unique perturbation combinations is $N_{\text{cond}}=N_{\text{chem}}\times N_{\text{cell}}\times N_{\text{dose}}$. We pre-calculate the $R_{\text{sim}}^{2}$  $\in R^{N_{\text{cond}}}$ between the mean expression of the control group and the mean expression of the perturbed group for each condition (details are provided in Section 3, available as supplementary data at *Bioinformatics* online). The dataset is thus defined as $\left(\text{cond},R_{\text{sim}}^{2}\right)$. Notably, $R_{\text{sim}}^{2}$ is used to determine whether a perturbation condition requires flow-based generation, whereas evaluation $R^{2}$ measures the agreement between model-generated expression profiles and the observed perturbed profiles in Stage 2.

#### 2.1.2 Condition encoding

For drug molecular encoding, we selected PubChem as the primary method, representing molecules as 881-dimensional binary vectors where each dimension indicates the presence or absence of a specific chemical substructure. To enable the model to generalize to unseen dosage levels and cell types, dosages are normalized using a reference dosage of 10 μM (i.e. $x_{\text{dose}}/10$) and transformed into an embedding of dimension $d_{\text{dose}}$ through a linear layer. Cell types are encoded using the mean gene expression profile of their corresponding control cells and transformed into a cell-type embedding of dimension $d_{\text{cell}}$. The resulting embeddings are concatenated with the drug representation to form the conditional input embedding $h_{\text{cond}}\in R^{d_{\text{drug}}+d_{\text{dose}}+d_{\text{cell}}}$.

#### 2.1.3 Response predictor architecture

We compared the performance of MLP and Transformer architectures. Results indicated that the ResMLP architecture is more suitable for this task, as excessively high model capacity can lead to overfitting on minority classes in imbalanced datasets. Our Response Predictor consists of multiple residual blocks, each containing a linear layer and a SiLU activation function. Notably, the final output ${\bar{R^{2}}}_{\text{sim}}$ is constrained within the range [−1,1] using an auxiliary output layer:


(1) $${\bar{R^{2}}}_{\text{sim}}=\text{tanh}(h)$$

It should be noted that while the theoretical range of $R_{\text{sim}}^{2}$ is (−∞,1], in practice, most of the observed minimum values are approximately −1. This bounded output significantly improves training stability compared to direct $R_{\text{sim}}^{2}$ regression.

#### 2.1.4 Imbalance training

To determine the optimal strategy for mitigating the severe data imbalance in the dataset, we evaluated several established techniques, including Oversampling, Balanced MSE, and GAILoss. Specifically, the Oversampling strategy operates at the data level by selectively duplicating minority-class instances—namely, those exhibiting low $R_{\text{sim}}^{2}$ values, which correspond to strong biological responses, to construct more balanced training batches. Alternatively, balanced MSE addresses imbalanced regression from a probabilistic perspective, modifying the standard mean squared error into a statistically principled, weighted loss function that accommodates skewed target distributions. GAI serves as an advanced refinement of this concept; it incorporates Gaussian priors to smooth the target label space, thereby reducing generalization errors in sparse, high-response regions.

Our empirical evaluations demonstrate that GAI consistently achieves superior performance compared to the other baseline strategies. Consequently, we integrate the GAI-based loss into our framework. First, GAI parameterizes the empirical training distribution $p_{\text{train}}(R_{\text{sim}}^{2})$ using a Gaussian Mixture Model (GMM):


(2) $$p_{\text{train}}(R_{\text{sim}}^{2})=\sum_{k=1}^{K}\pi_{k}N\left(R_{\text{sim}}^{2};\mu_{k},\Sigma_{k}\right)$$

where *K* is the number of Gaussian components, and $\pi_{k},\mu_{k},\Sigma_{k}$ represent the mixture weight, mean, and covariance matrix of the *k*-th component, respectively.

By substituting the GMM into the Balanced MSE objective, the product of two Gaussian distributions yields an unnormalized Gaussian, enabling a strictly analytical closed-form solution. Thus, the final GAI Loss function optimized in our first stage is formulated as:


(3) $$\begin{array}{c} L_{\text{GAI}}(R_{\text{sim}}^{2},{\bar{R^{2}}}_{\text{sim}})=\frac{{\left(R_{\text{sim}}^{2}-{\bar{R^{2}}}_{\text{sim}}\right)}^{2}}{2\sigma_{\text{noise}}^{2}}\;\;+\text{log}\;\sum_{k=1}^{K}\pi_{k}N\left({\bar{R^{2}}}_{\text{sim}};\mu_{k},\Sigma_{k}+\sigma_{\text{noise}}^{2}\right). \end{array}$$

where $\sigma_{\text{noise}}^{2}$ is a learnable parameter representing the noise variance.

### 2.2 Gene expression prediction stage

#### 2.2.1 Training data

For the generative stage, we exclusively retain perturbed gene expression samples where $R_{\text{sim}}^{2}$  <threshold, denoted as $x_{p}\in R^{N_{\text{gene}}}$.

#### 2.2.2 Condition encoding

The same drug encoding is reused in the prediction stage and transformed by a linear layer into $h_{\text{drug}}\in R^{d}$, where *d* denotes the target dimension. To support generalization to unseen dosages, the dosage value is normalized using a reference dosage of 10 µM (i.e. $x_{\text{dose}}/10$) and projected into a dosage embedding $h_{\text{dose}}\in R^{d}$ through a linear layer. Instead of using discrete cell-type embeddings, each cell type is represented by the mean expression profile of its corresponding control cells and transformed into a cell embedding $h_{\text{cell}}\in R^{d}$ via a linear layer. The time step *t* is encoded using an MLP layer to obtain the temporal embedding $h_{t}\in R^{d}$. These embeddings are combined through element-wise addition to form the final control signal:


(4) $$h_{\text{ctrl}}=h_{t}+h_{\text{cell}}+h_{\text{dose}}+h_{\text{drug}}.$$

#### 2.2.3 Flow matching model

While conventional approaches typically utilize pre-perturbation samples from the same cell type as the initial state, our empirical observations during training suggest that the marginal differences between pre- and post-perturbation profiles result in excessively slow model convergence. To address this, we instead employ a standard Gaussian distribution $x_{0}\in R^{N_{\text{gene}}}$ as the initial state for the generation process. The model defines a path between $x_{0}$ and the target distribution (perturbed expression $x_{p}$) by learning a time-dependent velocity field $v_{t}$ over the interval t∈[0,1].

We use $h_{\text{ctrl}}$ to modulate the velocity prediction via AdaLN. The control vector $h_{\text{ctrl}}$ is projected through a linear layer and split into four modulation parameters: scaling and gating factors for the Multi-Head Self-Attention block ($s_{\text{msa}},g_{\text{msa}}$) and the Feed-Forward Network ($s_{\text{mlp}},g_{\text{mlp}}$):


(5) $$s_{\text{msa}},g_{\text{msa}},s_{\text{mlp}},g_{\text{mlp}}=\text{Split}(\text{Linear}(h_{\text{ctrl}}))$$

To improve training stability by ensuring the model behaves as an identity mapping upon initialization, we apply the following transformations:


(6) $${\bar{g}}_{\text{msa}}=\text{tanh}(g_{\text{msa}}),\;{\bar{g}}_{\text{mlp}}=\text{tanh}(g_{\text{mlp}})$$

(7) $${\bar{s}}_{\text{msa}}=1+s_{\text{msa}},\;{\bar{s}}_{\text{mlp}}=1+s_{\text{mlp}}$$

Let MSA(·) represent the block of Multi-Head Self-Attention, FFN(·) represent Feed-Forward Network, and LN(·) represent Layer Norm block. The computation within each block is defined as follows:


(8) $$z_{t}=\text{ExpEmbedder}(x_{t})$$

(9) $$h_{t}=z_{t}+{\bar{g}}_{\text{msa}}\cdot \text{LN}(\text{MSA}(\text{LN}(z_{t})⊙{\bar{s}}_{\text{msa}}))$$

(10) $${\bar{h}}_{t}=h_{t}+{\bar{g}}_{\text{mlp}}\cdot \text{LN}(\text{FFN}(\text{LN}(h_{t})⊙{\bar{s}}_{\text{mlp}}))$$

where $z_{t}\in R^{N_{\text{gene}}\times d}$ is the hidden state at time *t*, and ExpEmbedder(·) independently projects each scalar gene-expression value $x_{t,g}$ into a *d*-dimensional gene token. Finally, the hidden state ${\bar{h}}_{t}\in R^{N_{\text{gene}}\times d}$ is projected back to the gene expression dimension to predict the velocity $v_{t}\in R^{N_{\text{gene}}}$:


(11) $$v_{t}=\text{Linear}({\bar{h}}_{t})$$

The sample at the next time step is updated as $x_{t+1}=x_{t}+v_{t}$. The final predicted expression ${\bar{x}}_{p}$ is obtained through multi-step ODE integration from t=0 to t=1, optimized using the MSE loss.

#### 2.2.4 Datasets and preprocessing

The single-drug perturbation datasets include SciPlex3 and McFarland. SciPlex3 contains 9-drug OOD split and two dosage splits, while McFarland includes five drug splits and three cell-type splits. The combinatorial drug perturbation dataset, ComboSciPlex, is a scRNA-seq subset derived from the combinatorial perturbation experiments in SciPlex3 and includes one drug combination split. More details are provided in Section 1, available as supplementary data at *Bioinformatics* online.

Gene expression values were normalized using log(x+1) and library size normali zation. We select the top 2000 Highly Variable Genes (HVGs) for SciPlex3 and McFarland dataset, and 1000 for ComboSciPlex dataset.

For each condition, we selected the top 50 differentially expressed genes (DEGs) as a subset for additional testing (detailed selection methods are provided in Sections 1 and 2, available as supplementary data at *Bioinformatics* online).

## 3 Experiments

### 3.1 Results and discussion

We benchmarked our method against state-of-the-art models on the SciPlex3, McFarland, and ComboSciPlex datasets, complemented by comprehensive ablation studies. The evaluation focuses on the accuracy of predicted gene expression values, the distribution alignment of generated samples under conditions with significant perturbation effects, and the biological validity of the generated responses through enrichment pathway analysis, overlap analysis, and perturbation direction consistency assessment. Furthermore, we systematically evaluate the computational efficiency of our method by analyzing training and inference costs in terms of both time and memory consumption. The detailed calculation procedures for all metrics are provided in Section 2, available as supplementary data at *Bioinformatics* online.

### 3.2 Generalization to unseen drugs

On the SciPlex3 dataset, we compared scBalFlow with chemCPA and biolord, using the optimal hyperparameters reported in their respective original studies. Details of the parameter selection strategy of scBalFlow are provided in Table 4, available as supplementary data at *Bioinformatics* online. We established an *unpredicted* baseline (Identity), which directly outputs the control group expression as the prediction. Furthermore, two variants were included for ablation: (i) **scBalFlow flow from ctrl**, an end-to-end flow matching model that generates expression directly from control samples and conditions, and (ii) **scBalFlow w/o threshold**, which trains on all conditions without filtering for weakly responsive samples in the second stage.

The drug perturbation prediction results on the SciPlex3 dataset evaluated on DEGs are presented in Fig. 3, while the evaluation results on the whole gene set are provided in Figs 2 and 3, available as supplementary data at *Bioinformatics* online. The advantage of scBalFlow becomes increasingly evident as the dosage increases, and it achieves significantly better performance than all baseline models at the highest dosage level. In terms of precision, at low dosages (e.g. 0.01 μM), most methods perform similarly to the baseline with high $R^{2}$ scores. However, as the dosage increases, the prediction error of traditional methods escalates, often falling below the baseline. In contrast, scBalFlow maintains superior performance, with its advantage over the baseline widening at higher doses. These results highlight the advantage of scBalFlow in handling perturbation imbalance and confirm the necessity of its two-stage design.

**Figure 3:**
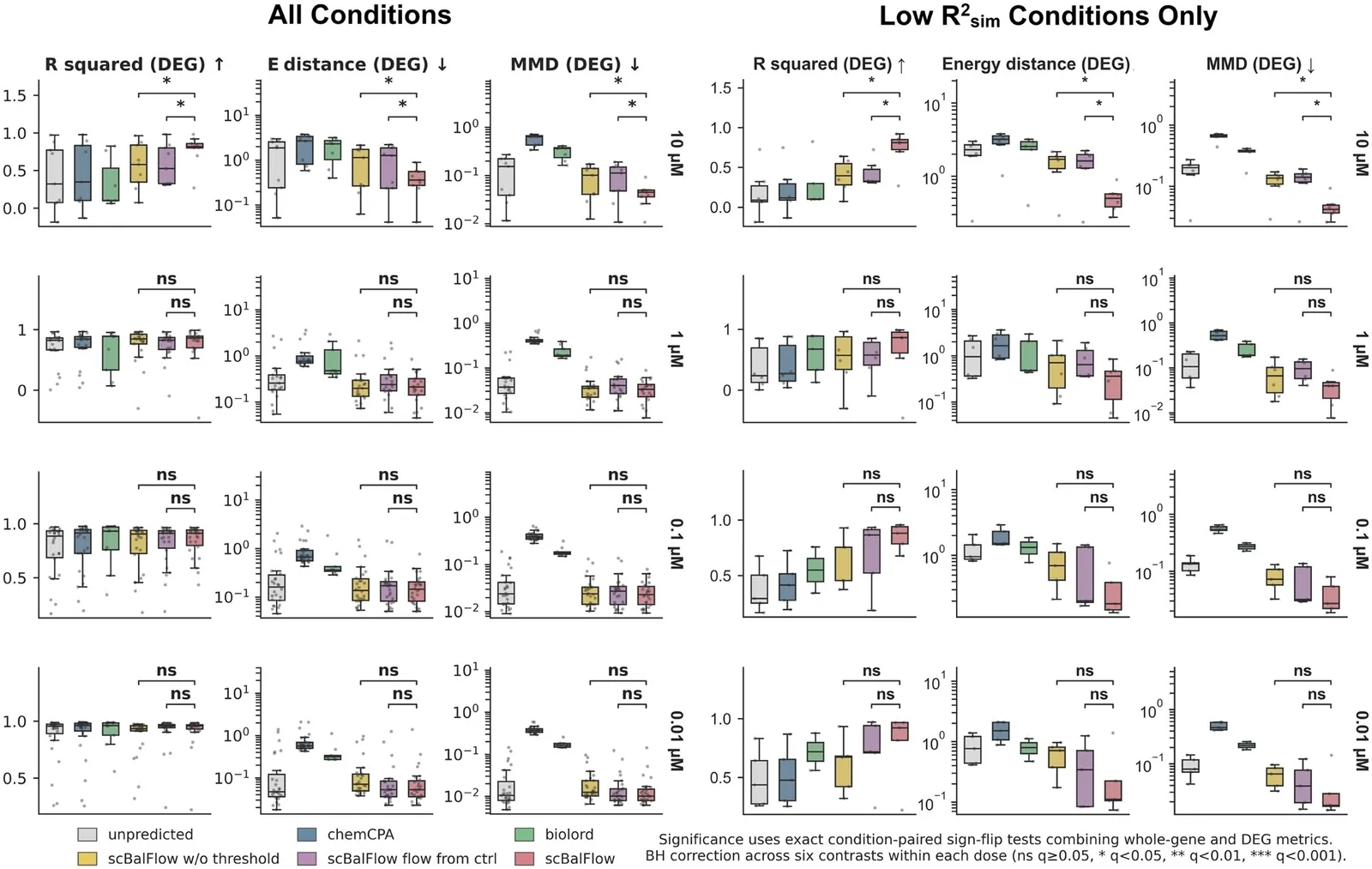
Performance comparison on the SciPlex3 dataset. The left three columns evaluate model performance across all perturbation conditions, whereas the right three columns focus only on highly responsive conditions with $R_{\text{sim}}^{2}$ below the predefined threshold. This comparison excludes the possibility that the advantage of scBalFlow is primarily attributed to directly sampling control-like cells rather than its generative capability. As the dosage increases (from 0.01 to 10.0 μM), the prediction errors of traditional methods escalate, often falling below the naive “unpredicted” baseline due to overfitting on weakly responsive majority samples. In contrast, scBalFlow generally achieved the best distributional metrics, with the largest and statistically significant improvements observed at 10 μM.

To visualize the ability of different models to capture perturbation-induced distribution shifts, we generated UMAP embeddings by integrating model predictions with the ground truth (Fig. 4). As shown in the UMAP projections, scBalFlow successfully recovers the heterogeneous response patterns across perturbation conditions. By leveraging flow matching in the second stage, scBalFlow generates distributions that closely align with the ground truth rather than collapsing toward the global expression mean. More crucially, baseline models are substantially affected by the perturbation imbalance and produce predictions concentrated around the control-like average profile, failing to generate highly responsive samples. In contrast, scBalFlow captures both weak and strong perturbation responses.

**Figure 4:**
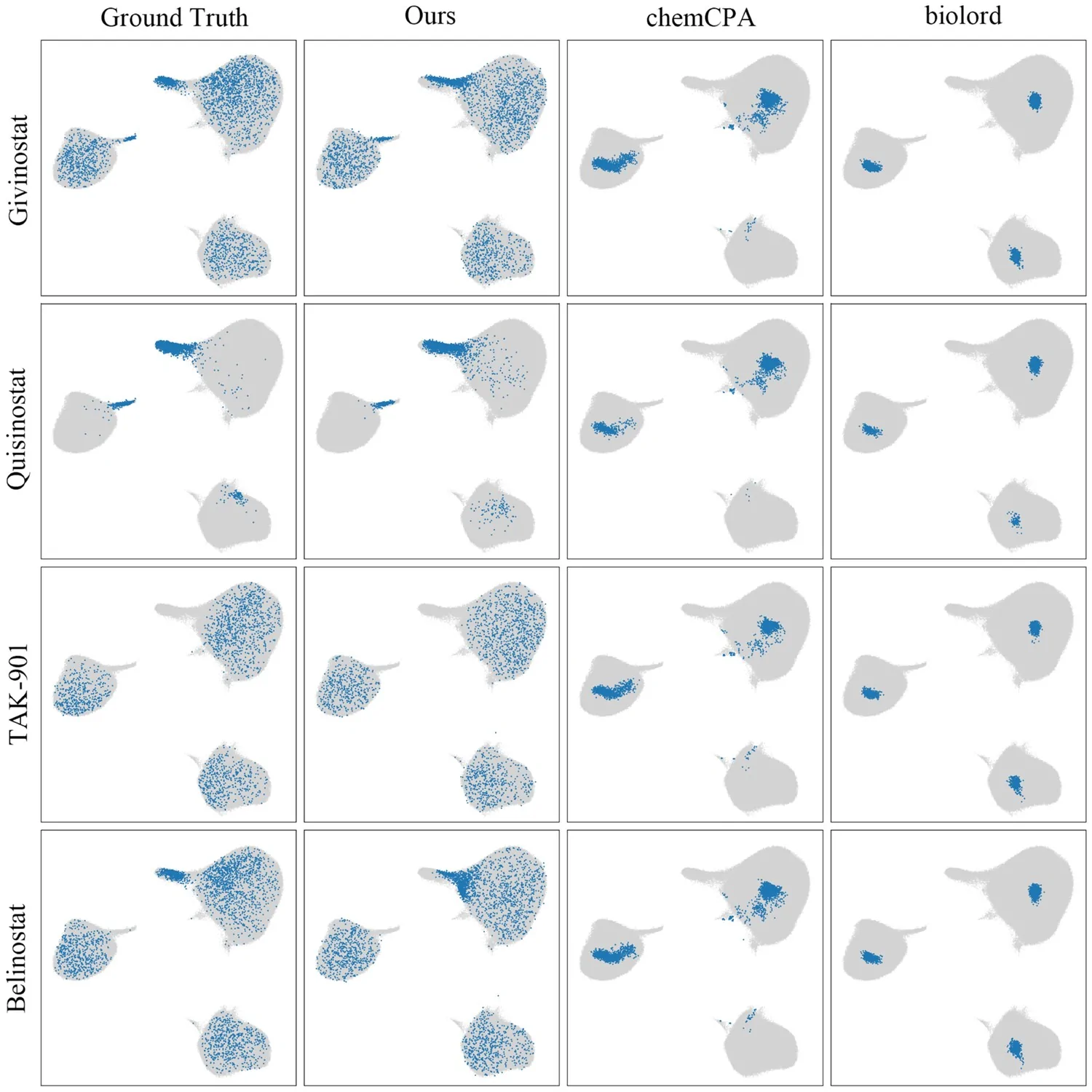
UMAP visualizations. Comparing the predicted single-cell distributions of scBalFlow, chemCPA, and biolord against the Ground Truth under four representative drug conditions, scBalFlow effectively captures varying perturbation dynamics: dose-sensitivity (Givinostat), profound universal shifts (Quisinostat), cell-type specificity (Belinostat), and accurately avoids spurious generations in weakly responsive scenarios (TAK-901).

On the McFarland dataset (Table 1), relative to chemCPA and biolord, scBalFlow reduced E-distance (DEGs) by 71% and 67%, respectively, and reduced MMD (DEGs) by 86% and 88%, respectively. T-tests further confirm that scBalFlow is significantly superior in distribution matching (P<.01). Performance across each split is visualized in Fig. 4, available as supplementary data at *Bioinformatics* online.

### 3.3 Biological relevance validations

Beyond quantitative prediction accuracy, we evaluated whether scBalFlow could recover biologically meaningful perturbation responses through DEG recovery and pathway-level validation on matched OOD conditions.

On SciPlex3, we analyzed 22 matched OOD conditions (Table 2). scBalFlow achieved the highest average top-k DEG overlap and substantially improved DEG direction recovery, with a direction consistency score of 0.826 compared with 0.597 for biolord and 0.391 for chemCPA.

**Table 1:** Performance evaluation on the McFarland dataset.^a^

Metric	scBalFlow (ours)	chemCPA	biolord
$R^{2}$	0.916 ± 0.042	0.785 ± 0.060^b^	0.907 ± 0.026
E-distance	1.145 ± 0.486	5.304 ± 0.986^b^	4.136 ± 0.449^b^
MMD	0.073 ± 0.025	0.449 ± 0.069^b^	0.639 ± 0.040^b^
$R^{2}$ (DEGs)	0.894 ± 0.061	0.798 ± 0.067	0.857 ± 0.061^b^
E-distance (DEGs)	0.654 ± 0.285	2.256 ± 0.439^b^	1.979 ± 0.324^b^
MMD (DEGs)	0.070 ± 0.023	0.495 ± 0.055^b^	0.583 ± 0.030^b^

a Results are reported as mean  ±  SD across five-fold cross-validation. The best performance is highlighted in bold.

b Indicates P<.01 (Welch’s *t*-test) compared to scBalFlow.

**Table 2:** SciPlex3 overlap and differential-expression direction agreement.^a^

Model	Overlap@50	Overlap@100	Overlap@200	DE direction match
chemCPA	0.0564 ± 0.0398	0.1332 ± 0.0355	0.2467 ± 0.0549	0.3905 ± 0.1600
biolord	0.0918 ± 0.0519	0.0950 ± 0.0421	0.1512 ± 0.0679	0.5970 ± 0.0537
scBalFlow	0.1127 ± 0.0658	0.2177 ± 0.0841	0.2792 ± 0.1037	0.8258 ± 0.0623

a Values are mean  ±  SD across evaluated perturbations. The best performance is highlighted in bold.

We further performed pre-ranked gene set enrichment analysis using MSigDB Hallmark pathways. As shown in Table 3, scBalFlow achieved higher pathway-level agreement, with a mean NES Spearman correlation of 0.699, pathway-direction accuracy of 0.832, and top-10 pathway overlap of 0.586, substantially exceeding existing baselines. Detailed visualizations are provided in Figs 5 and 6, available as supplementary data at *Bioinformatics* online.

**Table 3:** SciPlex3 pathway-enrichment prediction metrics.^a^

Model	NES Spearman ρ	Direction accuracy	Top-10 overlap
chemCPA	0.111 ± 0.179	0.627 ± 0.210	0.300 ± 0.120
biolord	0.132 ± 0.221	0.569 ± 0.143	0.355 ± 0.163
scBalFlow (ours)	0.699 ± 0.249	0.832 ± 0.157	0.586 ± 0.149

a Values are mean  ±  SD across evaluated conditions. The best performance is highlighted in bold.

To evaluate combinatorial perturbation prediction, we compared scBalFlow with scDFM on the ComboSciPlex dataset (Table 1, available as supplementary data at *Bioinformatics* online). scBalFlow achieved higher top-100 and top-200 DEG overlap, while slightly lower direction consistency, indicating competitive performance despite the smaller scale and weaker imbalance of this dataset.

### 3.4 Generalization to unseen dosages and cell types

We evaluated scBalFlow under unseen dosage generalization using two SciPlex3 dose-OOD splits, where either 1 or 10 μM conditions were held out. As shown in Table 4 and Fig. 9, available as supplementary data at *Bioinformatics* online, scBalFlow achieved better performance than chemCPA on all whole-gene metrics and on DEG-based distributional metrics, whereas chemCPA obtained a higher $R^{2}$ on DEGs. Importantly, the same performance pattern was observed across both held-out dose settings, with scBalFlow consistently yielding lower Energy Distance and MMD values. biolord was excluded from this analysis because it jointly encodes drug and dosage as a combined categorical representation.

**Table 4:** Evaluation of OOD dose prediction on the SciPlex3 dataset.^a^

Metric	scBalFlow (ours)	chemCPA
$R^{2}$	0.943 ± 0.016	0.891 ± 0.002
E-distance	0.139 ± 0.047	1.183 ± 0.021
MMD	0.012 ± 0.003	0.487 ± 0.003
$R^{2}$ (DEGs)	0.915 ± 0.018	0.958 ± 0.004
E-distance (DEGs)	0.108 ± 0.034	0.352 ± 0.007
MMD (DEGs)	0.017 ± 0.006	0.176 ± 0.002

a Results are summarized across two dose-OOD splits, with 1 μM or 10 μM held out. The best performance is highlighted in bold.

We also assessed cell-type generalization using three held-out-cell folds from the McFarland dataset. As shown in Table 5 and Fig. 10, available as supplementary data at *Bioinformatics* online, scBalFlow and scGen achieved comparable $R^{2}$ performance, while scBalFlow consistently produced lower Energy Distance and MMD values across all three held-out cell-type splits. Models relying on learned categorical representations, including chemCPA and biolord, were not evaluated in this setting because they cannot represent unseen cell identities.

**Table 5:** Evaluation of OOD cell-type prediction on the McFarland dataset.^a^

Metric	scBalFlow (ours)	scGen
$R^{2}$	0.883 ± 0.015	0.888 ± 0.009
E-distance	1.488 ± 0.113	2.633 ± 0.112
MMD	0.085 ± 0.008	0.152 ± 0.010
$R^{2}$ (DEGs)	0.893 ± 0.002	0.897 ± 0.002
E-distance (DEGs)	0.672 ± 0.032	1.021 ± 0.046
MMD (DEGs)	0.074 ± 0.005	0.158 ± 0.014

a Results are summarized across three held-out-cell-type splits. The best performance is highlighted in bold.

### 3.5 Ablation study

To optimize the Stage-1 perturbation predictor, we compared three imbalance-handling strategies (Oversampling, Balanced MSE, and GAI Loss) with the standard MSE baseline (Fig. 11, available as supplementary data at *Bioinformatics* online). Standard MSE collapsed to predicting $R_{\text{sim}}^{2}\approx 1$ for all samples under severe imbalance. Oversampling showed limited generalization due to overestimation of response intensity, while Balanced MSE produced unstable low-$R_{\text{sim}}^{2}$ predictions. In contrast, GAI Loss achieved a better balance between detecting active perturbations and maintaining accurate response estimation.

We further evaluated the sensitivity of GMM components, initial noise scale, noise learning rate, Stage-1 learning rate, and early-stopping patience across 30 configurations and 12 OOD tasks (Fig. 12, available as supplementary data at *Bioinformatics* online). The initial noise scale was the most influential factor, with $\sigma_{0}=0.3$ achieving the lowest validation MSE, whereas other parameters showed limited effects. The selected model hyperparameters are summarized in Table 4, available as supplementary data at *Bioinformatics* online.

We evaluated SciPlex3 thresholds of 1.0 (no filtering), 0.9, 0.8, 0.7, and 0.6. As shown in Table 5, available as supplementary data at *Bioinformatics* online, the threshold 0.8 gives the best joint result: $R^{2}=0.891$, Energy Distance =0.234 and MMD =0.0167.

### 3.6 Computational cost analysis

We benchmarked computational efficiency on a single NVIDIA RTX 4090 GPU, including model size, peak GPU memory usage, and inference time (Table 2 and Fig. 13, available as supplementary data at *Bioinformatics* online). Overall, computational efficiency depends on representation and workload design. While CellFlow benefits from compact features, scBalFlow achieves scalability through effective workload reduction in the generative stage.

Despite operating in a higher-dimensional gene-expression space, scBalFlow achieves favorable inference efficiency through imbalance-aware routing rather than feature compression. As shown in Table 3, available as supplementary data at *Bioinformatics* online, threshold filtering substantially reduces the number of conditions and cells processed by Stage-2 without changing the generator architecture, thereby avoiding unnecessary generation for near-control perturbations.

## 4 Discussion

scBalFlow addresses the severe imbalance in single-cell perturbation datasets by separating response estimation from expression generation. This design prevents the flow model from being dominated by weakly responsive conditions and reduces computation by applying generative modeling only to conditions predicted to exhibit substantial transcriptomic changes. Ablation and threshold-sensitivity analyses further demonstrate that response-aware routing improves both prediction accuracy and efficiency.

However, the current hard routing strategy relies on dataset-specific threshold selection, and $R_{\text{sim}}^{2}$ may overlook subtle but biologically meaningful responses. Future work could develop uncertainty-aware soft routing and incorporate complementary signals, such as pathway activity or differential-expression patterns, to better capture weak responses while maintaining the efficiency benefits of routing.

## 5 Conclusion

In this study, we identified severe response imbalance as an important challenge that can limit the ability of generative models to capture strong perturbation responses in single-cell drug perturbation datasets. To address this challenge, we proposed scBalFlow, a decoupled two-stage framework that separates response estimation from gene expression generation. The first stage employs a GAI strategy to estimate perturbation response intensity, while the second stage applies Flow Matching selectively to conditions with substantial transcriptional changes.

Comprehensive evaluations on SciPlex3, McFarland, and ComboSciPlex demonstrate that scBalFlow consistently improves distributional alignment across different perturbation generalization scenarios. The improvements are observed across multiple evaluation metrics and held-out settings, indicating the robustness of the proposed response-aware modeling strategy. The proposed response-aware routing strategy also reduces unnecessary computation associated with near-control perturbations. These results suggest that explicitly modeling response imbalance provides a promising direction for scalable and biologically meaningful single-cell perturbation prediction.

## Supplementary Material

btag682_Supplementary_Data

## Data Availability

The data underlying this article are available. All links are provided under the Availability and implementation section of the Abstract.
