# Supplementary material for: scBalFlow: a staged flow matching framework for imbalanced single-cell drug perturbation prediction
Source: Bioinformatics. 2026 Sep 15;42(9):btag682. doi: 10.1093/bioinformatics/btag682 (PMC13619003; doi:10.1093/bioinformatics/btag682)
Supplement: Supplementary file 1 [file btag682_supplementary_data.pdf]

Supplementary Information

scBalFlow: A Staged Flow Matching Framework for Imbalanced Single-Cell Drug Perturbation Prediction

Section S1: Datasets Introduction

- **SciPlex3** is a large-scale, high-throughput single-cell drug perturbation dataset and remains one of the most widely used benchmarks in the field. It contains approximately 560,000 samples, covering 188 drugs, 4 dosages, and 3 cell types.

  Following (Lotfollahi et al.,2023), we held out 9 specific drugs (Hesperadin, TAK-901, Dacinostat, Givinostat, Belinostat, Quisinostat, Alvespimycin, Tanespimycin, and Flavopiridol) as an Out-of-Distribution (OOD) test set.

  Gene expression values were normalized using $\log(x + 1)$ and library size normalization. We select the top 2,000 Highly Variable Genes (HVGs). Additionally, we identified 50 Differentially Expressed Genes (DEGs) by comparing the expression profiles of the control and perturbed groups using all available conditions in the complete dataset. The top-50 DEG list is generated separately for each perturbation condition. Specifically, a condition is defined as a unique combination of cell type, drug, and dosage. For each condition, we calculated the mean expression profile of the perturbed cells and compared it with the mean expression profile of the matched control cells from the same cell type. Genes were ranked according to the absolute difference between these two mean profiles, and the top 50 genes were selected as condition-specific DEGs for downstream evaluation.

- **McFarland** is a single-cell drug perturbation dataset which comprises approximately 100,000 samples across 171 cell types, 6 dosages, and 13 drugs. Preprocessing followed the same protocol as SciPlex3. We also implemented a 5-fold cross-validation split to ensure the stability and robustness of our findings.

- **ComboSciPlex** is a scRNA-seq subset of the combinatorial experiment of SciPlex3. It contains approximately 63,000 samples, covering 32 drug combinations, 1 dosage, and 1 cell type.

  Following scDFM and CellFlow, we held out 7 specific drug combinations (Panobinostat+Crizotinib, Panobinostat+Curcumin, Panobinostat+SRT1720, Panobinostat+Sorafenib, SRT2104+Alvespimycin, control+Alvespimycin, and control+Dacinostat).

Section S2: Evaluation Metrics

Perturbation similarity score (R^2_{sim}). To quantify the global transcriptional response strength of each perturbation condition, we defined a similarity score between the perturbed and matched control expression profiles. For a given perturbation condition, we first identified the corresponding cell type and calculated the mean expression vector of control cells from the same cell type:

$$\boldsymbol{\mu}_{\text{ctrl}} = \frac{1}{N_{\text{ctrl}}} \sum_{i=1}^{N_{\text{ctrl}}} \boldsymbol{x}_i^{\text{ctrl}},$$

where N_{ctrl} denotes the number of control cells and $\boldsymbol{x}_i^{\text{ctrl}}$ represents the gene expression vector of the i-th control cell. Similarly, the mean expression vector of the perturbed population was calculated as:

$$\boldsymbol{\mu}_{\text{pert}} = \frac{1}{N_{\text{pert}}} \sum_{i=1}^{N_{\text{pert}}} \boldsymbol{x}_i^{\text{pert}}.$$

The perturbation similarity score was defined as the coefficient of determination between the control and perturbed mean expression profiles, where the perturbed mean profile was treated as the predicted value and the control mean profile was treated as the reference value:

$$y_{\text{true}} = \boldsymbol{\mu}_{\text{ctrl}}, \qquad y_{\text{pred}} = \boldsymbol{\mu}_{\text{pert}}.$$

Specifically, R^2_{sim} was calculated as:

$$R^2_{\text{sim}} = 1 - \frac{\sum_{g=1}^{G} (\mu_{\text{ctrl},g} - \mu_{\text{pert},g})^2}{\sum_{g=1}^{G} (\mu_{\text{ctrl},g} - \overline{\mu}_{\text{ctrl}})^2},$$

where G denotes the number of genes and $\overline{\mu}_{\text{ctrl}}$ represents the mean expression value across genes in the control mean profile. A value close to 1 indicates that the perturbed population remains globally similar to the matched control population, whereas lower values indicate stronger transcriptomic deviation.

Prediction accuracy metrics. For each perturbation condition, the predicted cell population was compared with the experimentally observed perturbed population. The coefficient of determination was calculated between their mean expression profiles:

$$R^2 = R^2(\boldsymbol{\mu}_{\text{true}}, \boldsymbol{\mu}_{\text{pred}}),$$

where $\boldsymbol{\mu}_{\text{true}}$ and $\boldsymbol{\mu}_{\text{pred}}$ represent the mean expression vectors of the observed and predicted cells, respectively.

Energy distance. To evaluate distributional similarity beyond mean expression accuracy, we calculated the energy distance between the observed and predicted single-cell distributions. Given two expression distributions X and Y, the energy distance is defined as:

$$D_E(X, Y) = 2\mathbb{E}\left[\|X - Y\|_2\right] - \mathbb{E}\left[\|X - X'\|_2\right] - \mathbb{E}\left[\|Y - Y'\|_2\right],$$

where X' and Y' are independent samples from the corresponding distributions. Lower energy distance indicates better agreement between predicted and observed cellular distributions.

Maximum mean discrepancy (MMD). The maximum mean discrepancy was used to quantify distributional differences in a reproducing kernel Hilbert space. The MMD is defined as:

$$\text{MMD}(X, Y) = \mathbb{E}_{x,x'}[k(x, x')] + \mathbb{E}_{y,y'}[k(y, y')] - 2\mathbb{E}_{x,y}[k(x, y)],$$

where $k(\cdot, \cdot)$ denotes a Gaussian radial basis function kernel:

$$k(x, y) = \exp(-\gamma \|x - y\|_2^2).$$

Following our implementation, MMD was averaged across multiple kernel bandwidths with

$$\gamma \in \{2, 1, 0.5, 0.1, 0.01, 0.005\}.$$

Lower MMD values indicate closer agreement between predicted and observed distributions.

Differentially expressed gene (DEG) recovery. To evaluate whether models recovered perturbation-specific transcriptional changes, we compared the differentially expressed genes between predicted and observed perturbations. For each condition, genes were ranked according to the absolute difference between the perturbed and matched control mean expression profiles:

$$\delta_g = \mu_g^{\text{pert}} - \mu_g^{\text{ctrl}}, \qquad \Delta_g = |\delta_g|.$$

The top-k DEG overlap was calculated as:

$$\text{Overlap@}k = \frac{|G_{\text{true}}^k \cap G_{\text{pred}}^k|}{k},$$

where G_{true}^k and G_{pred}^k denote the top-k ranked DEGs from the observed and predicted perturbation responses, respectively.

The DEG direction consistency was calculated as:

$$\text{DirectionMatch} = \frac{1}{|\mathcal{G}|} \sum_{g \in \mathcal{G}} \mathbb{I}\left[\text{sign}\left(\delta_g^{\text{true}}\right) = \text{sign}\left(\delta_g^{\text{pred}}\right)\right],$$

where

$$\delta_g^{\text{true}} = \mu_{\text{pert},g}^{\text{true}} - \mu_{\text{ctrl},g}, \qquad \delta_g^{\text{pred}} = \mu_{\text{pert},g}^{\text{pred}} - \mu_{\text{ctrl},g}.$$

and $\mathcal{G}$ is the condition-specific top-50 DEGs identified from the observed perturbed and matched-control profiles.

Pathway enrichment evaluation. Pathway-level perturbation responses were evaluated using pre-ranked gene set enrichment analysis (GSEA) against the MSigDB Hallmark gene sets. For each perturbation condition, genes were ranked according to the mean expression difference between perturbed cells and matched control cells:

$$s_g = \mu_g^{\text{pert}} - \mu_g^{\text{ctrl}}.$$

The ranked gene list was analyzed using pre-ranked GSEA to obtain normalized enrichment scores (NES) for each pathway. The predicted pathway responses were compared with observed responses using NES Spearman correlation:

$$\rho_{\text{NES}} = \rho(NES_{\text{true}}, NES_{\text{pred}}).$$

The NES direction accuracy was calculated as:

$$\text{Direction Accuracy} = \frac{1}{P} \sum_{p=1}^{P} \mathbb{I}(\text{sign}(NES_p^{\text{true}}) = \text{sign}(NES_p^{\text{pred}})),$$

where P is the number of evaluated pathways.

Finally, top-k pathway overlap was calculated as:

$$\text{Pathway Overlap@}k = \frac{|P_{\text{true}}^k \cap P_{\text{pred}}^k|}{k}.$$

Statistical significance testing on SciPlex3. For the SciPlex3 ablation analysis, statistical significance was assessed separately at each dosage using an exact one-sided paired sign-flip test. The cell type–drug condition was used as the paired unit, and only conditions available for both scBalFlow and the corresponding ablation model were included. For each metric family, the whole-gene and DEG-restricted measurements were jointly considered. Let $d_{c,k}$ denote the performance improvement of scBalFlow over an ablation model for condition c and component k, where $k \in \{\text{all}, \text{DEG}\}$. The direction of the difference was defined such that a positive value consistently indicated better performance by scBalFlow:

$$d_{c,k} = \begin{cases} M_{c,k}^{\text{scBalFlow}} - M_{c,k}^{\text{ablation}}, & \text{for } R^2, \\ M_{c,k}^{\text{ablation}} - M_{c,k}^{\text{scBalFlow}}, & \text{for Energy Distance and MMD.} \end{cases}$$

To prevent the component with the larger numerical scale from dominating the combined statistic, each component was normalized by the root mean square of its paired differences:

$$r_k = \sqrt{\frac{1}{N} \sum_{c=1}^{N} d_{c,k}^2}.$$

A condition-level family score was then calculated as

$$s_c = \frac{1}{2} \sum_{k \in \{\text{all}, \text{DEG}\}} \frac{d_{c,k}}{r_k},$$

and the observed test statistic was the mean standardized improvement:

$$T_{\text{obs}} = \frac{1}{N} \sum_{c=1}^{N} s_c.$$

Under the null hypothesis that the paired differences were symmetrically distributed around zero, the signs of the condition-level scores were independently flipped while the whole-gene and DEG components of each condition were kept together. The exact one-sided p-value was computed as

$$p = \frac{1}{2^N} \sum_{\epsilon \in \{-1,+1\}^N} \mathbb{I}\left[\frac{1}{N} \sum_{c=1}^{N} \epsilon_c s_c \geq T_{\text{obs}} \right],$$

where the alternative hypothesis was that scBalFlow achieved better performance than the ablation model. Within each dosage, the resulting p-values from the six comparisons, comprising three metric families and two ablation models, were adjusted using the Benjamini–Hochberg procedure.

Statistical significance testing on McFarland. For the McFarland comparisons, statistical significance was assessed using a two-sided Welch's independent-samples t-test, which does not assume equal variances between the two groups. Let $\{x_i\}_{i=1}^{n_x}$ and $\{y_j\}_{j=1}^{n_y}$ denote the evaluation scores obtained by scBalFlow and a comparison model, respectively. The test statistic was calculated as

$$t = \frac{\bar{x} - \bar{y}}{\sqrt{\frac{s_x^2}{n_x} + \frac{s_y^2}{n_y}}},$$

where $\bar{x}$ and $\bar{y}$ are the sample means and s_x^2 and s_y^2 are the corresponding unbiased sample variances. The degrees of freedom were approximated using the Welch–Satterthwaite equation:

$$\nu = \frac{\left(\frac{s_x^2}{n_x} + \frac{s_y^2}{n_y} \right)^2}{\frac{\left(s_x^2/n_x\right)^2}{n_x - 1} + \frac{\left(s_y^2/n_y\right)^2}{n_y - 1}}.$$

The two-sided p-value was computed as

$$p = 2\,\Pr(T_\nu \geq |t_{\text{obs}}|),$$

under the null hypothesis that the two models had equal population mean performance.

Supplementary Figure S1: Relationship between global similarity and biological response magnitude

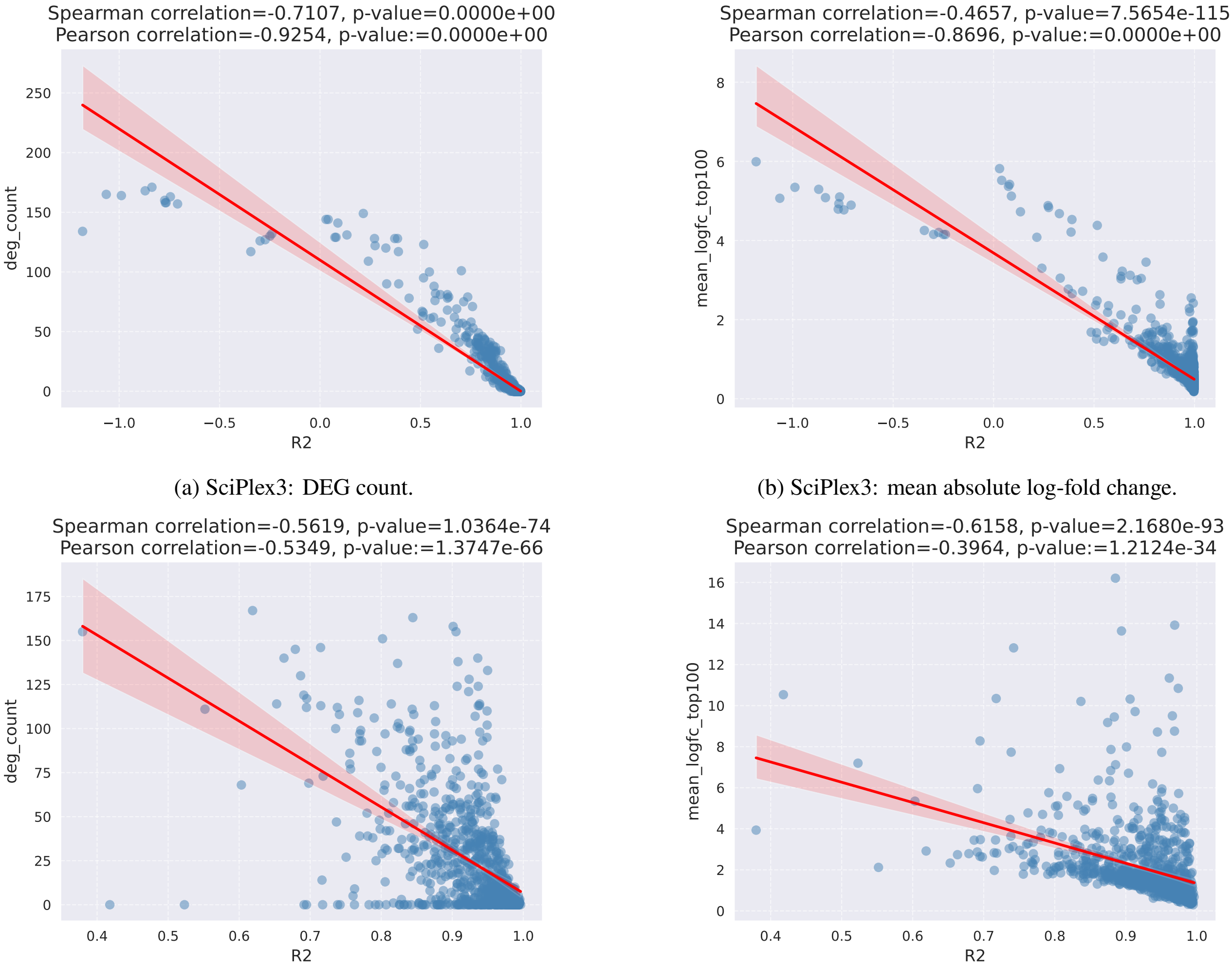

(a) SciPlex3: DEG count.

(b) SciPlex3: mean absolute log-fold change.

(c) McFarland: DEG count.

(d) McFarland: mean absolute log-fold change.

Figure S1: Lower condition-level control–perturbation R^2_{sim} is associated with more DEGs and larger expression changes. Spearman correlations are -0.711 and -0.466 for SciPlex3 and -0.562 and -0.616 for McFarland. The weaker association and residual dispersion in McFarland reinforce that R^2_{sim} is a global routing proxy rather than a deterministic biological label.

Supplementary Figure S2: Whole-gene SciPlex3 performance

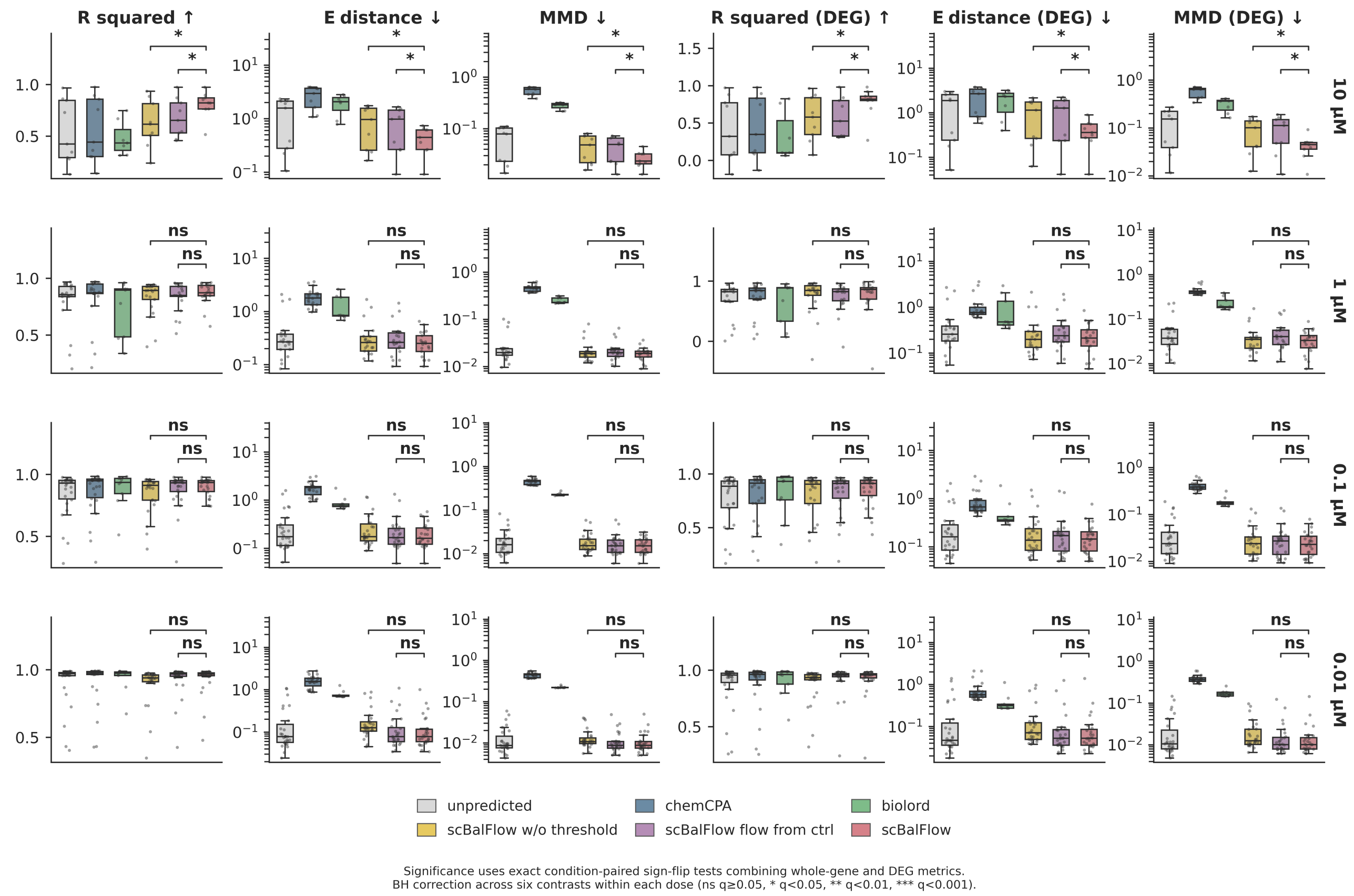

Figure S2: SciPlex3 evaluation on all 2,000 genes. Rows correspond to physical concentrations of 10, 1, 0.1 and 0.01 μM. The ablation previously called "one step" is reported as "flow from ctrl" to state explicitly that its initial distribution consists of randomly sampled matched-control cells rather than Gaussian noise. Significance brackets summarize the exact condition-paired metric-family sign-flip tests described in Section S2

Supplementary Figure S3: Evaluation restricted to routed responsive conditions

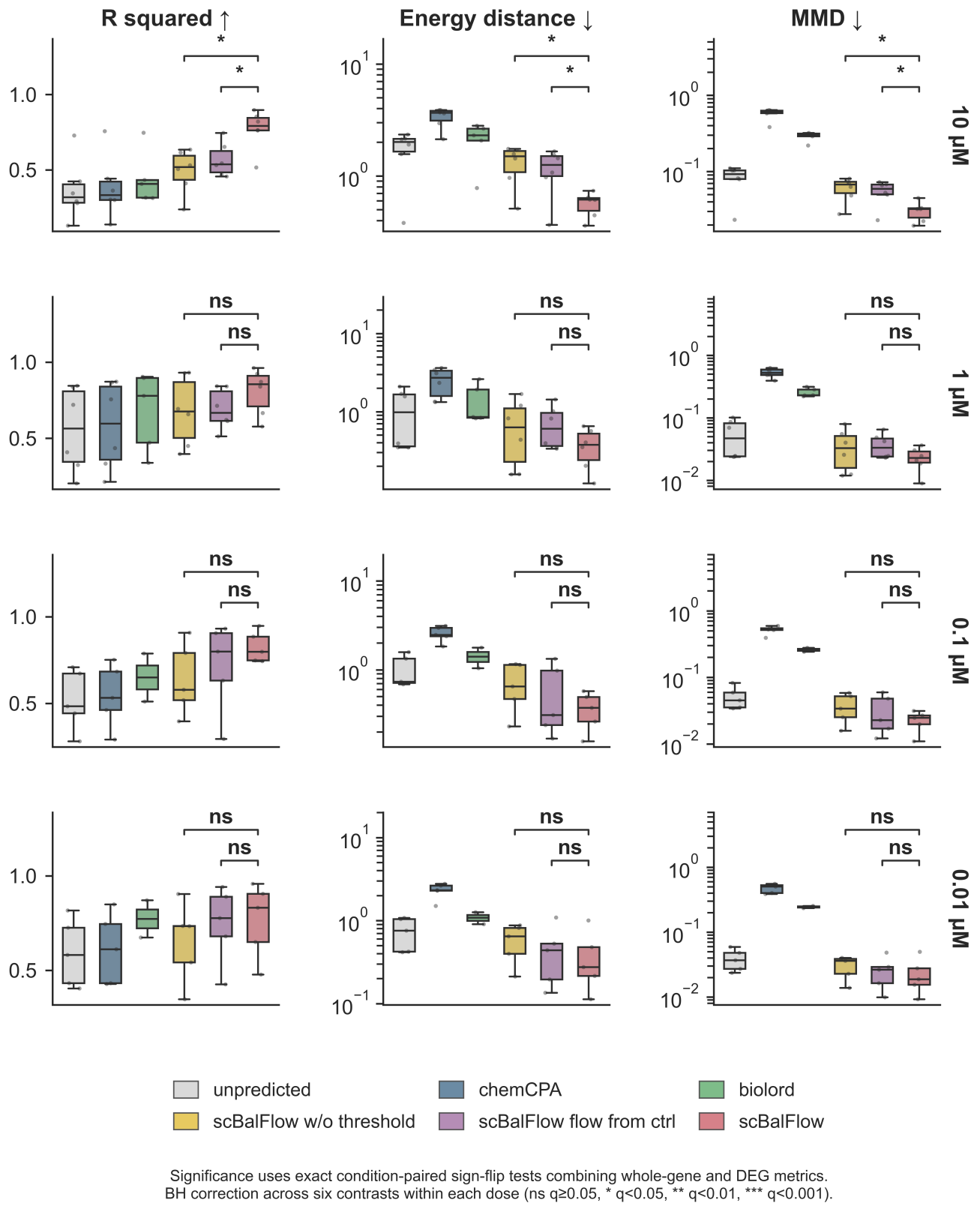

(a) All 2,000 genes.

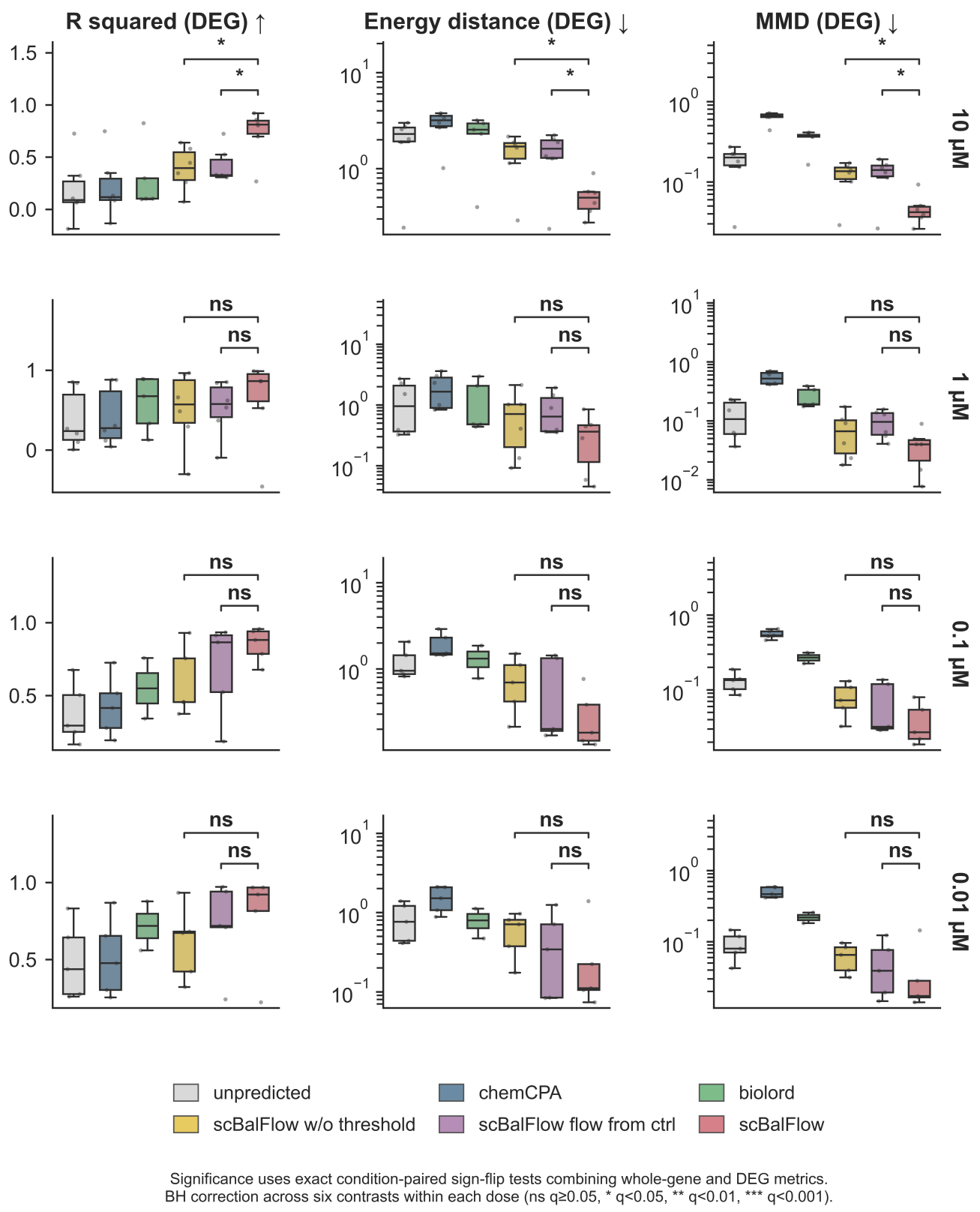

(b) Condition-specific top-50 DEG sets.

Figure S3: Performance after restricting evaluation to conditions below the global-similarity routing threshold. This analysis tests whether the full-model result is explained only by matched-control sampling for globally near-control conditions.

Supplementary Figure S4: Drug-level split on McFarland Dataset

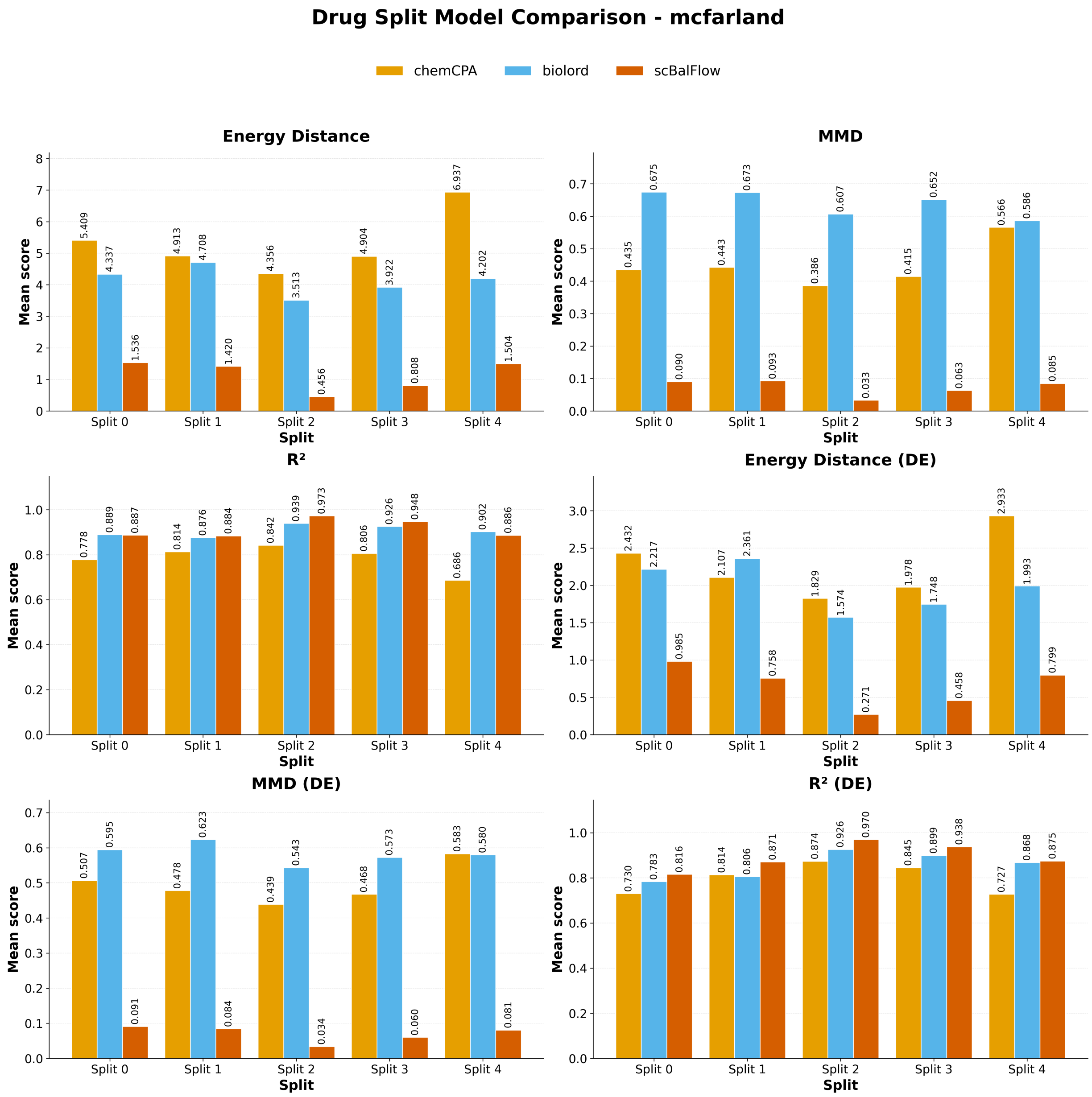

Figure S4: McFarland drug-level cross-validation summary.

Supplementary Figures S5–S6: Pathway-level evaluation

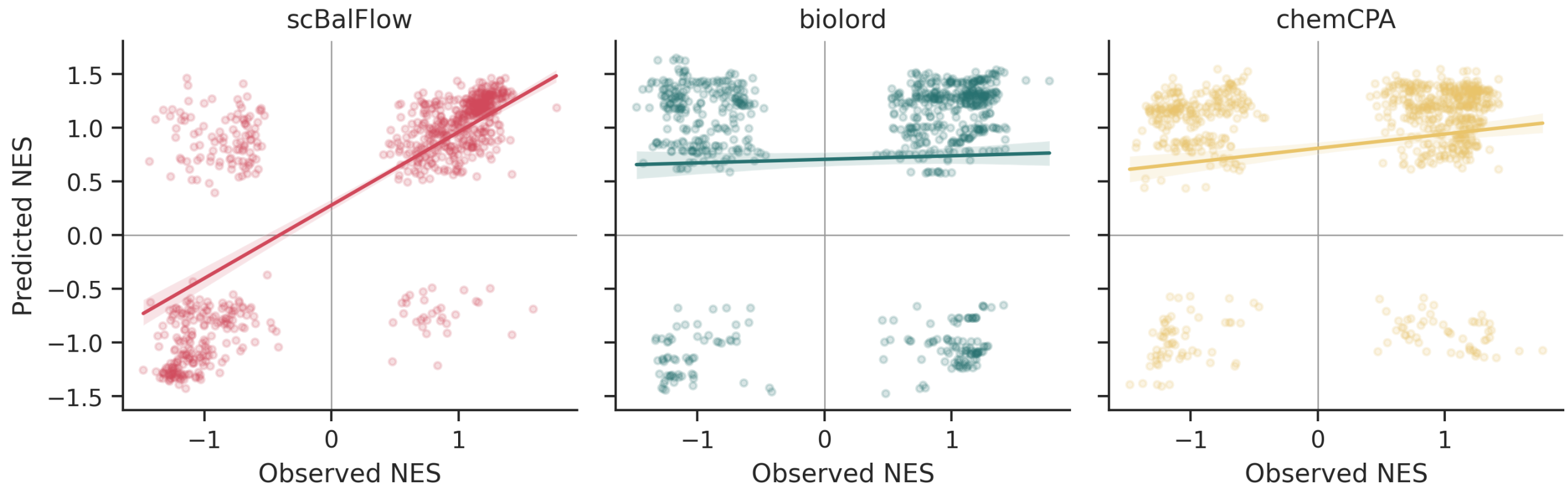

Figure S5: Observed versus predicted Hallmark normalized enrichment scores across matched SciPlex3 OOD conditions.

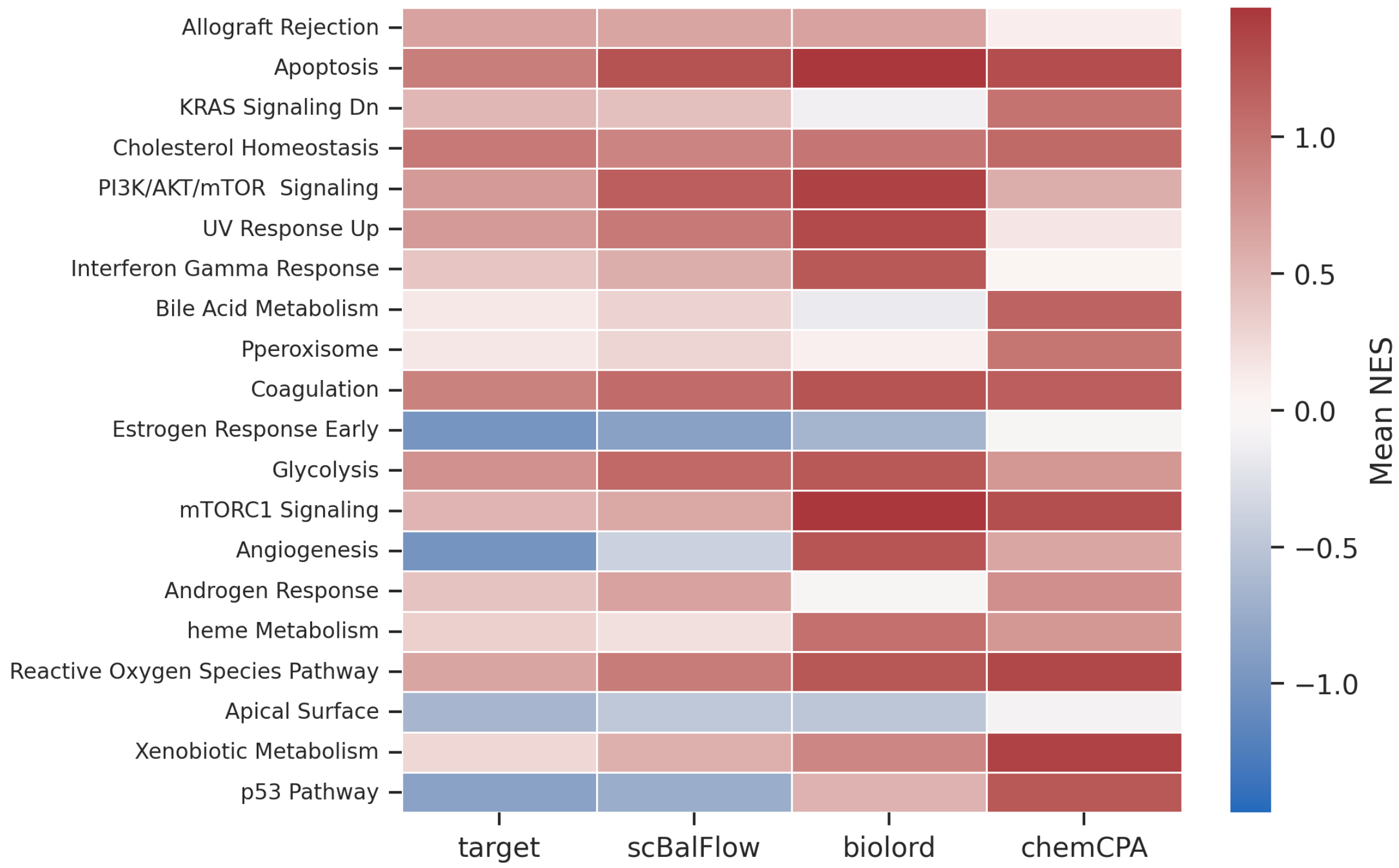

Figure S6: Mean normalized enrichment scores for representative Hallmark pathways. Columns show observed targets and model predictions.

Supplementary Table S1: Combinatorial Perturbation Prediction on the ComboSciPlex dataset

Table S1: ComboSciPlex split-level prediction metrics. Values are mean ± standard deviation across evaluated perturbations.

Model	Overlap@50	Overlap@100	Overlap@200	DE direction match
scBalFlow	0.1400 ± 0.0738	**0.1933 ± 0.0971**	**0.2258 ± 0.1169**	0.9091 ± 0.1558
scDFM	**0.1457 ± 0.1031**	0.1686 ± 0.1152	0.1793 ± 0.1229	**0.9126 ± 0.0980**

Supplementary Figures S7–S8: Routing-threshold sensitivity

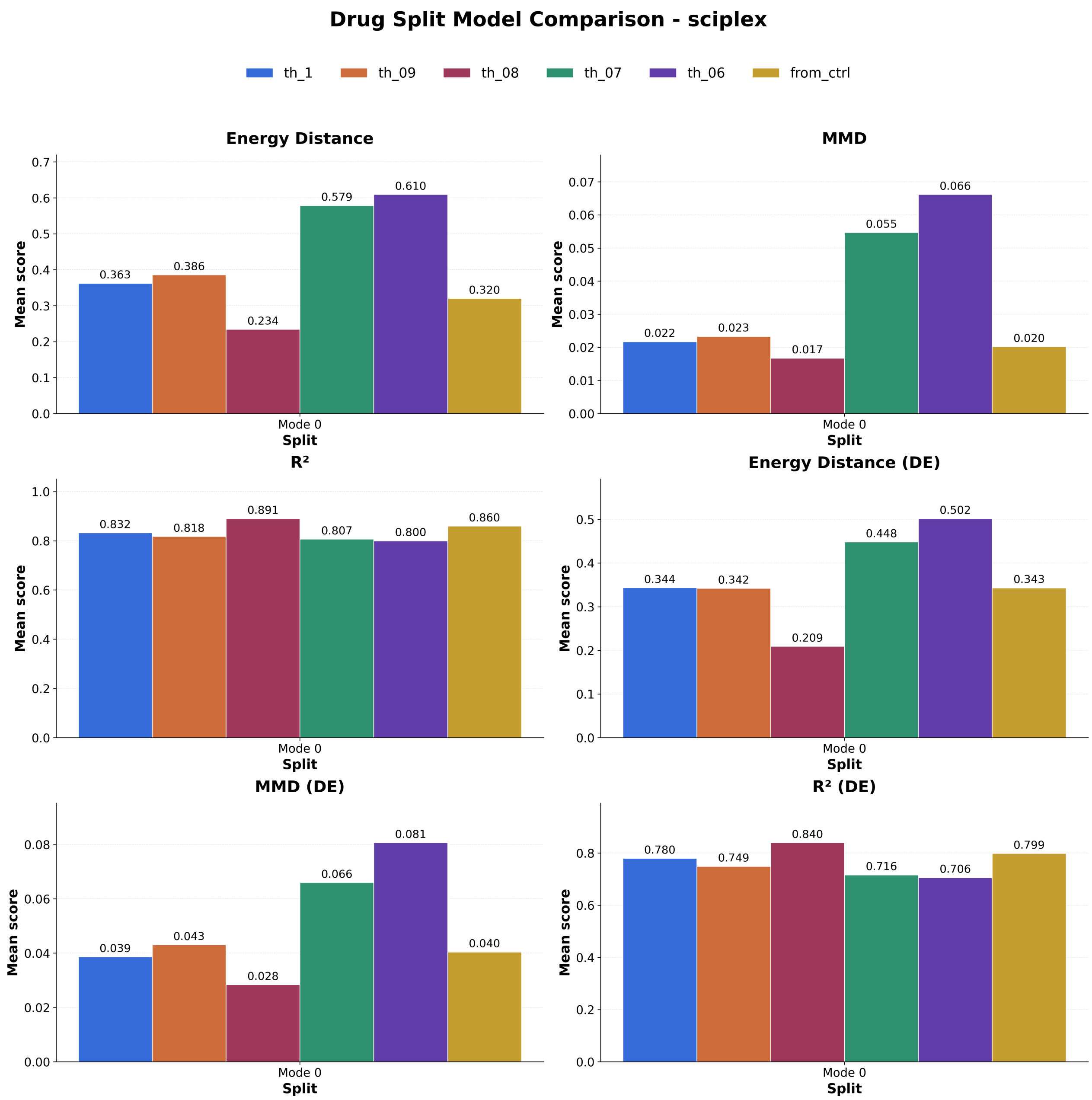

Figure S7: SciPlex3 performance under alternative routing thresholds.

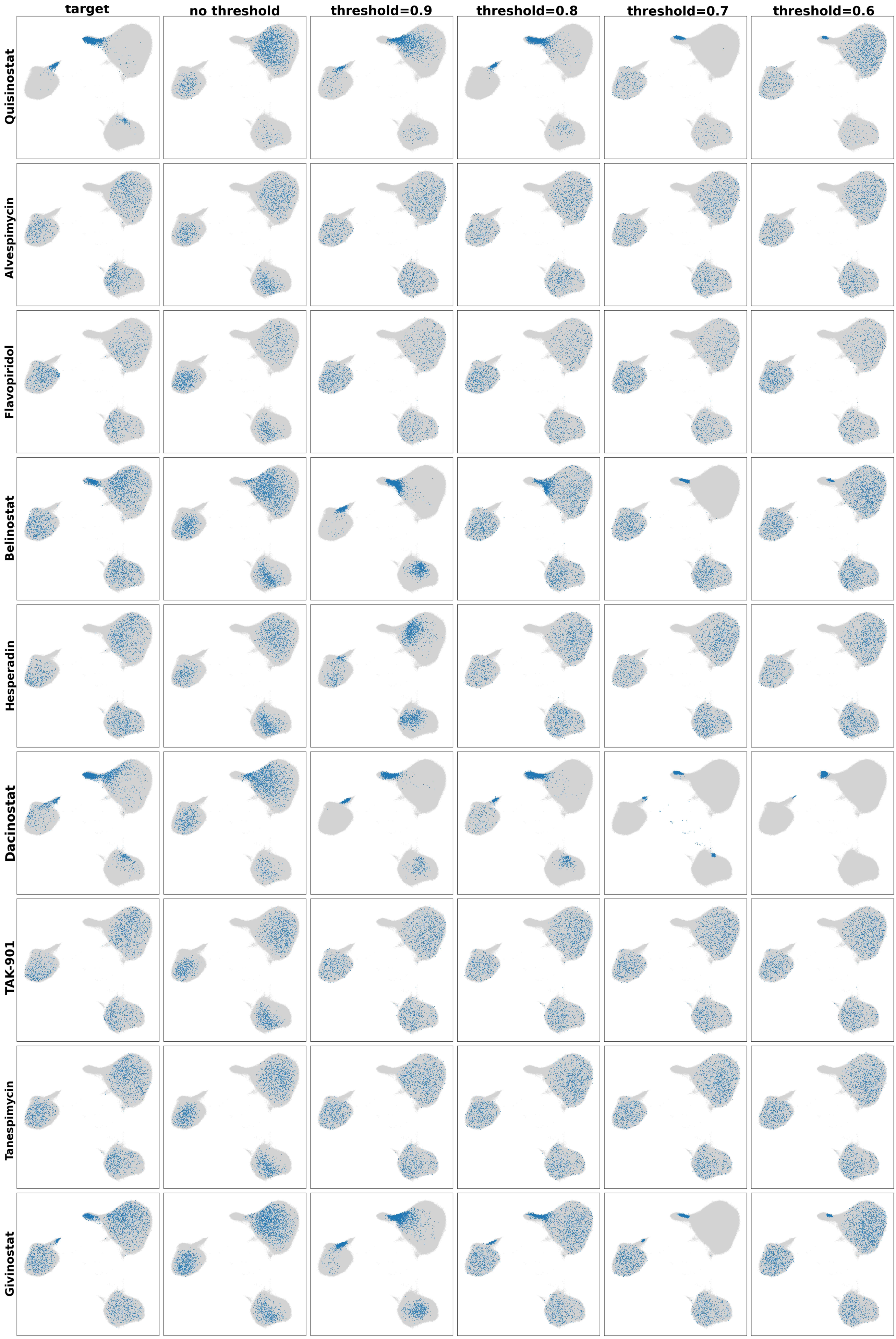

Figure S8: Illustrative routing-threshold sensitivity. Very low thresholds bypass an increasing fraction of the observed response, whereas no filtering sends globally near-control conditions through the generator.

Supplementary Figures S9–S10: Dose- and cell-type-OOD prediction

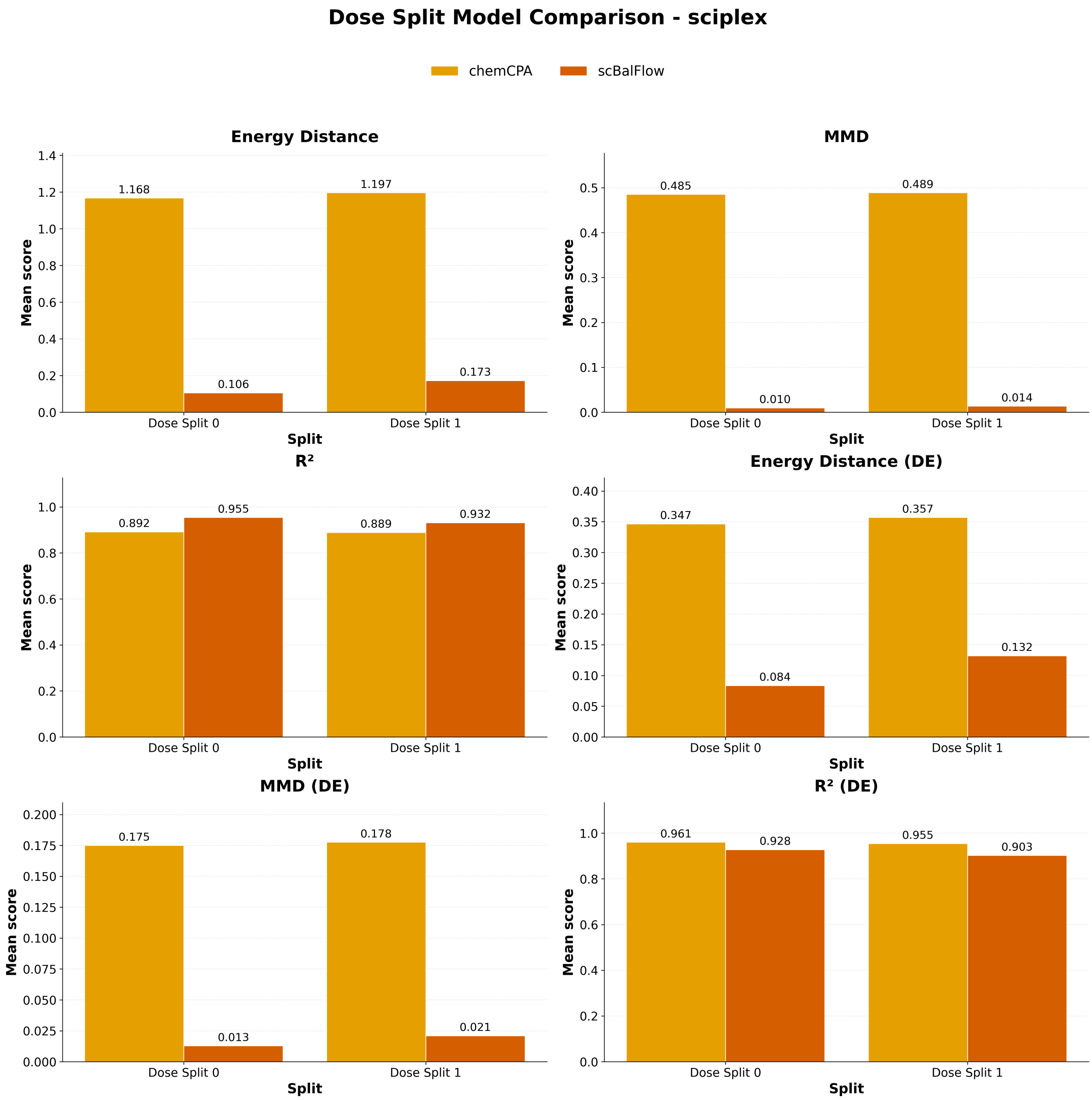

Figure S9: SciPlex3 dose-OOD comparison. Dose split 0 holds out 1 μM and dose split 1 holds out 10 μM.

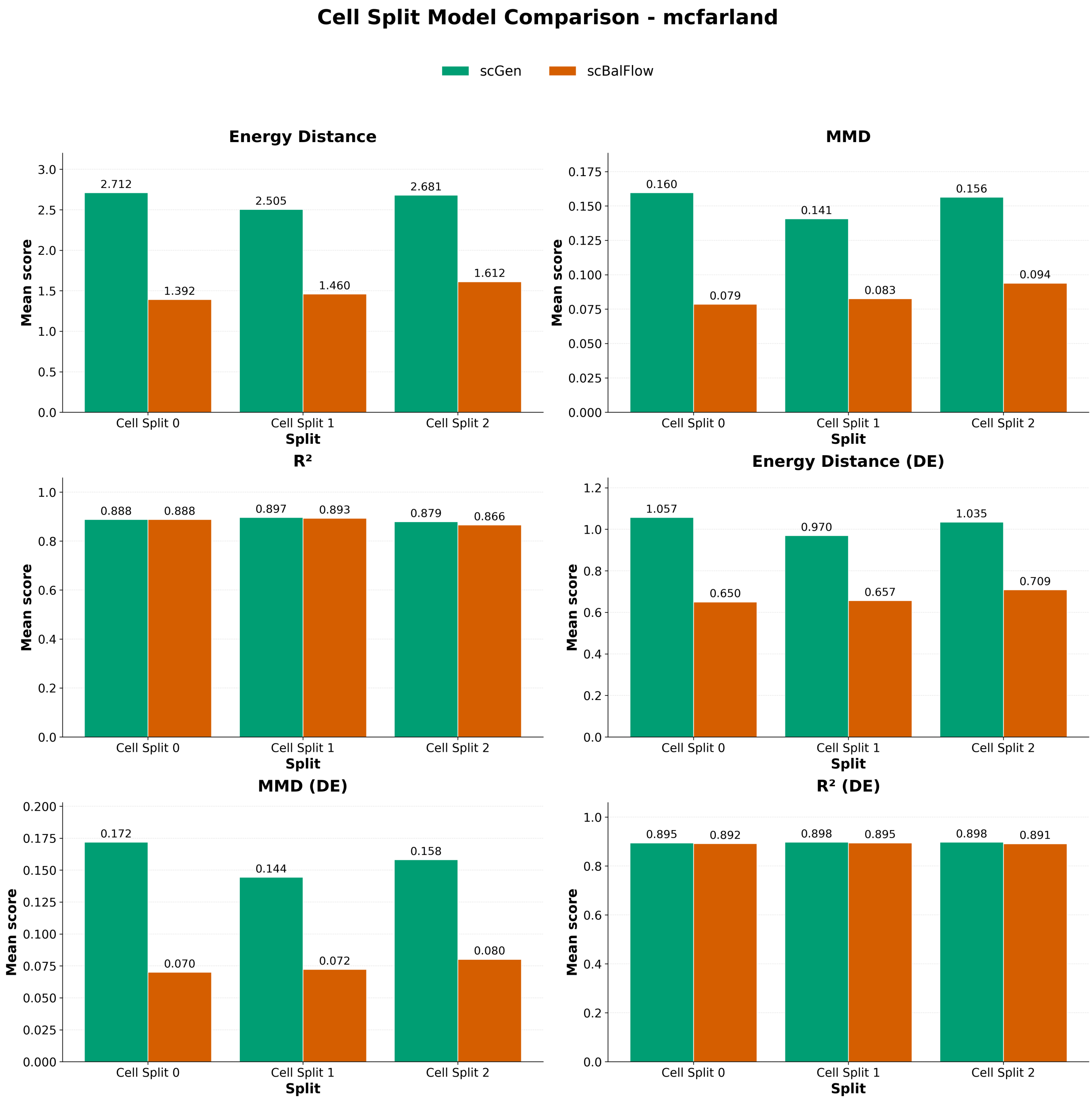

Figure S10: McFarland held-out-cell comparison across three folds. scBalFlow and scGen have comparable mean-profile R^2, while scBalFlow has lower Energy Distance and MMD.

Supplementary Figure S11: Stage-1 imbalance strategies

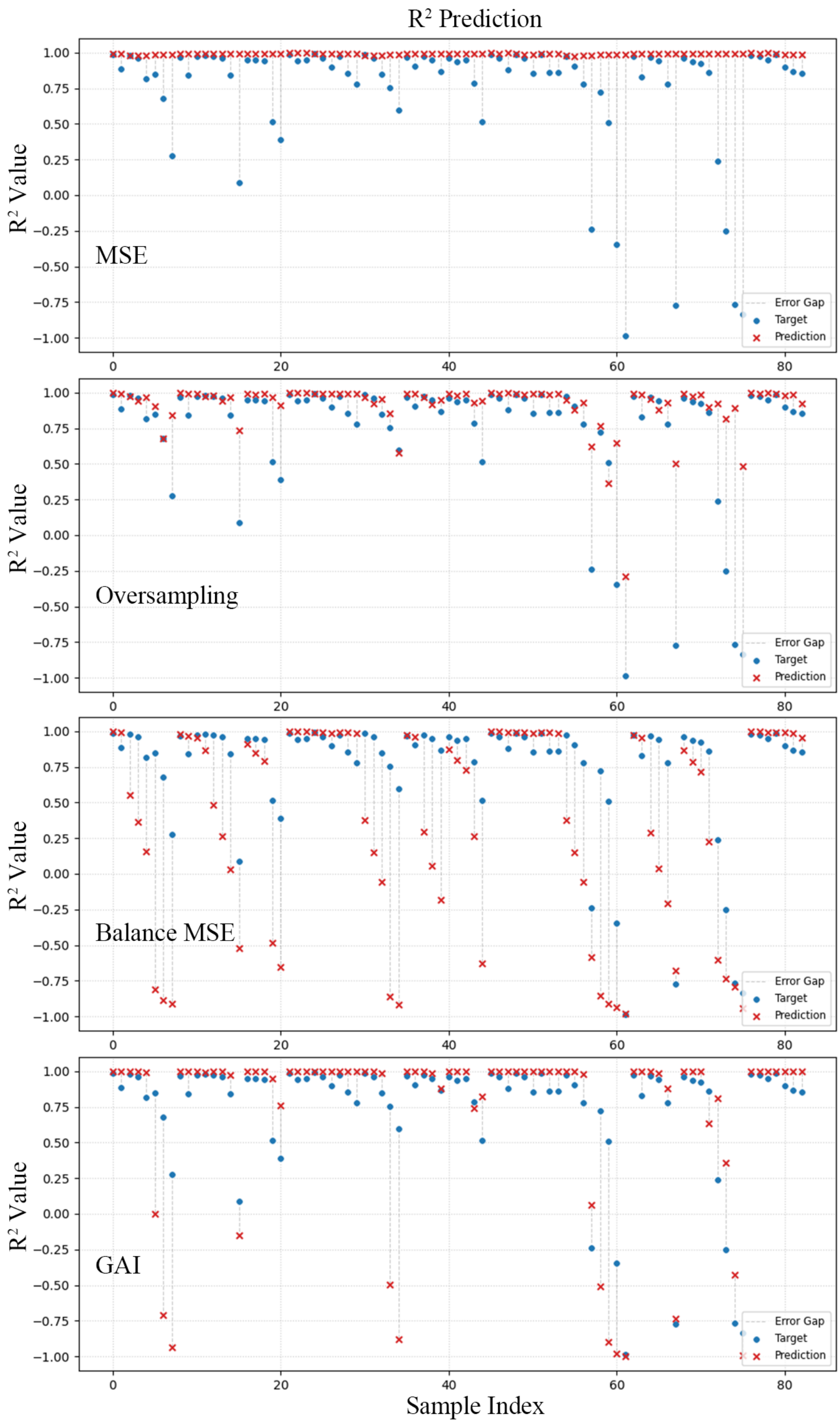

Figure S11: Comparison of standard MSE, oversampling, Balanced MSE and GAI Loss for Stage-1 R^2_{sim} prediction. GAI provides the best validation fit among the tested strategies without the strong majority collapse or over-correction observed for the alternatives.

Supplementary Tables S2–S3: Computational benchmark and routing workload

Table S2: Raw computational benchmark. Stage 1 is excluded because it contains only approximately two thousand condition-level examples and a three-layer residual MLP, making its cost negligible relative to Stage-2. CellFlow models a compact 100-dimensional PCA representation using a six-layer MLP with a hidden dimension of 4,096, whereas scDFM adopts a Transformer architecture with 1,000 input features. In contrast, scBalFlow directly models 2,000 gene features using a Transformer-based generator. For CellFlow, ^{a}1,451 cells denotes the full control-cell input used for one-condition prediction rather than a conventional mini-batch.

Model	Feat.	Train batch	Train iter.	Train time (min)	Train mem (MiB)	Infer batch	Infer mem (MiB)	Infer time (ms/cell)
CellFlow	100	1,024	300,000	2,100	4,530 peak	$1,451^{a}$	2,734 peak	92
scDFM	1,000	12	30,000	150	18,238 peak	96	3,638 peak	4242
scBalFlow (no th.)	2,000	16	≈101,460	180	4,011/4,144 (alloc./res.)	256	2,377/3,766 (alloc./res.)	26
scBalFlow ($th = 0.8$)	2,000	16	≈16,480	16	4,011/4,144 (alloc./res.)	256	2,377/3,766 (alloc./res.)	11

Table S3: Stage-2 workload under alternative SciPlex3 routing thresholds. Inference times are reported as minutes:seconds from the recorded runs.

Threshold	Stage-2 training cells	Inference time
1.0	541,130	493
0.9	31,057	260
0.8	16,471	201
0.7	11,108	62
0.6	8,972	43

Routing reduces the number of Stage-2 batches and therefore total training and inference time. It does not reduce peak generator memory because the architecture, feature dimension and per-batch tensor shapes are unchanged. CellFlow achieves high throughput using 100 PCA features and a six-layer 4096-dimensional MLP. scDFM uses a Transformer but includes high-dimensional feed-forward, W_{qkv} and fusion projections. These differences motivate reporting raw settings rather than claiming that one generator is intrinsically cheapest.

Supplementary Figure S12: GAI Loss Hyperparameter Sensitivity

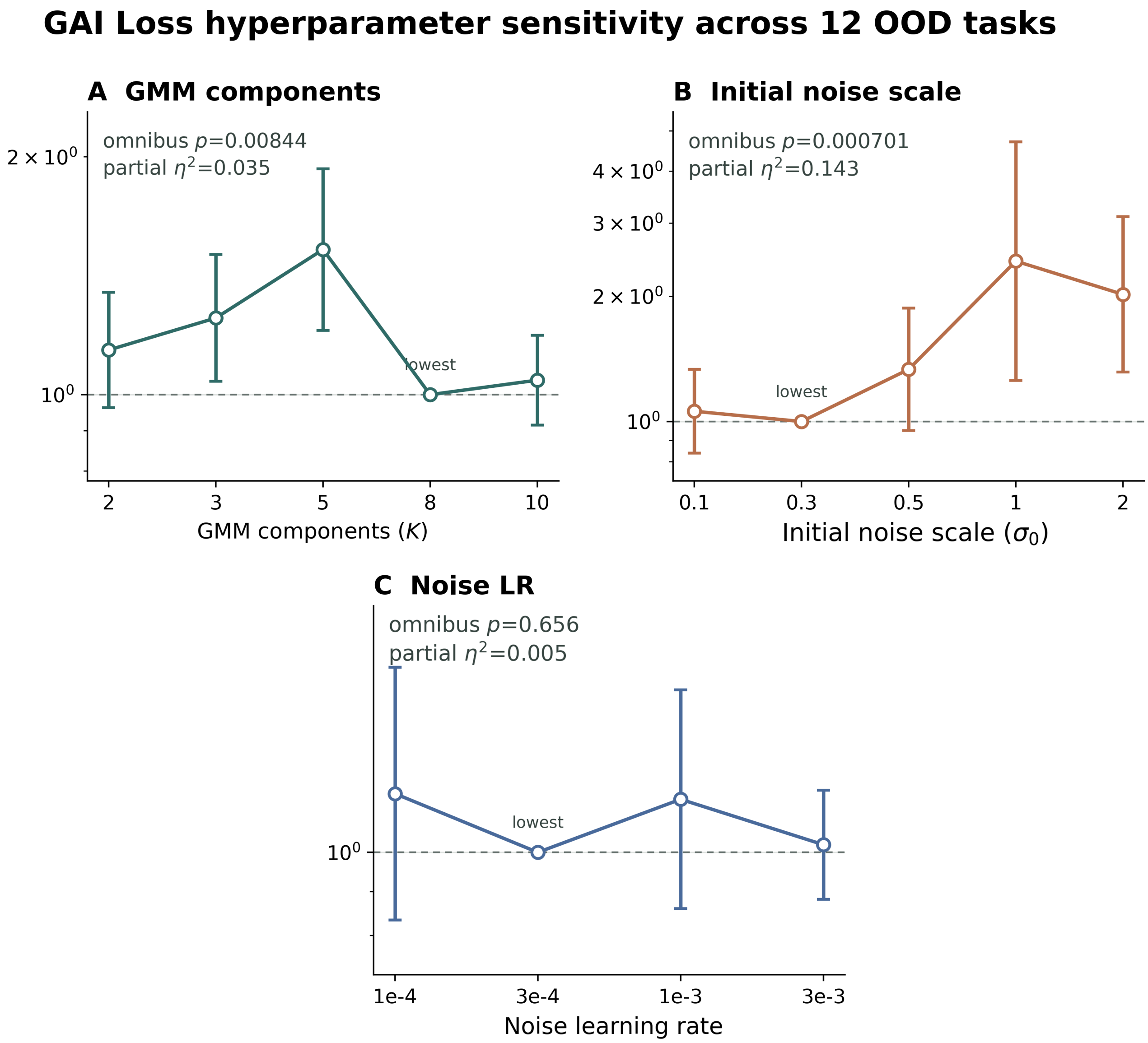

Figure S12: **GAI Loss hyperparameter sensitivity.** Ratios are estimated from log(validation MSE), adjusting for task and all other hyperparameters; lower is better. Error bars: task-clustered 95% CI.

Supplementary Figure S13: Computational Efficiency Comparison

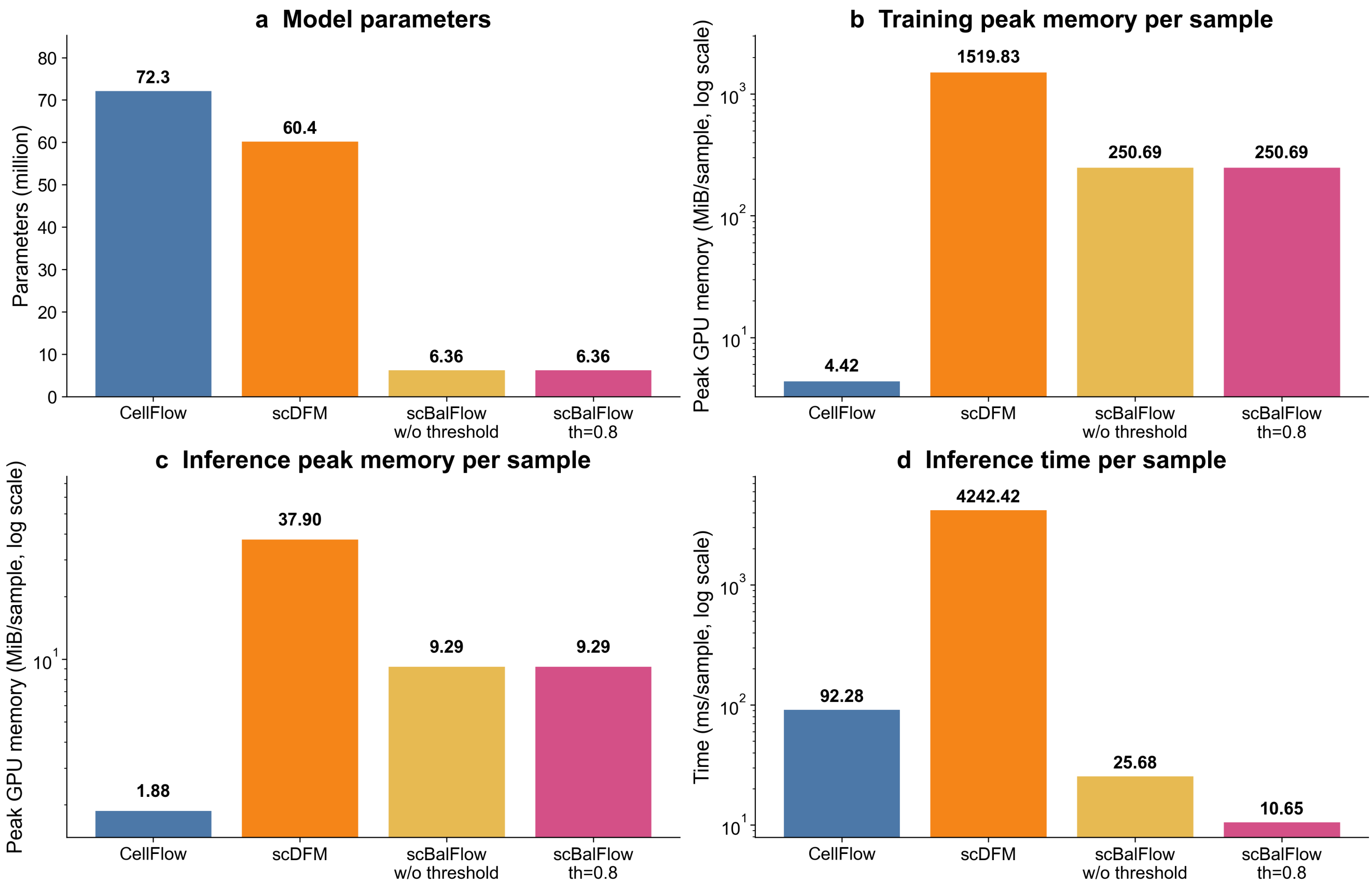

Figure S13: **Computational efficiency comparison.** Comparison of model parameter counts, peak training and inference GPU memory per sample, and inference time per generated sample. Results for scBalFlow are reported with and without threshold-based routing.

Supplementary Table S4: Model Configuration

Table S4: Model hyperparameters used in scBalFlow experiments. Dataset-specific parameters are reported separately.

Category	Hyperparameter	Value
**General settings**		
	Search strategy	Random search
	Number of search trials	30
	Random seed	42
	Search objective	Validation MSE
	Early stopping min Δ	1×10^{-6}
	Validation strategy	Held-out samples from both classes
	DataLoader workers	4
	Evaluation batch size	1024
	Gradient clipping norm	5.0
	EMA decay	0.999
**Hyperparameter search space**		
	Stage-1 learning rate	$\{10^{-4}, 3 \times 10^{-4}, 10^{-3}, 3 \times 10^{-3}\}$
	Stage-1 patience	$\{3, 5, 10\}$
	GMM components	$\{2, 3, 5, 8, 10\}$
	Initial noise σ	$\{0.1, 0.3, 0.5, 1.0, 2.0\}$
	Noise learning rate	$\{10^{-4}, 3 \times 10^{-4}, 10^{-3}, 3 \times 10^{-3}\}$
**SciPlex3**		
	Learning rate (Stage 2)	5×10^{-4}
	Epochs	200
	Batch size	16
	Stage-1 batch size	16
	Training data ratio	0.7
	Dropout	0.2
	Hidden dimensions	[1024, 512, 256]
	Drug embedding dimension	1024
	Routing threshold	0.8
	Maximum dose	10
**ComboSciPlex**		
	Learning rate (Stage 2)	5×10^{-4}
	Epochs	200
	Batch size	16
	Stage-1 batch size	4
	Training data ratio	0.7
	Dropout	0.1
	Hidden dimensions	[1024, 512, 256]
	Drug embedding dimension	1024
	Routing threshold	0.9
**McFarland**		
	Learning rate (Stage 2)	5×10^{-3}
	Epochs	200
	Batch size	8
	Stage-1 batch size	8
	Training data ratio	0.7
	Dropout	0.2
	Hidden dimensions	[1024, 512, 256]
	Drug embedding dimension	1024
	Routing threshold	0.9

Supplementary Table S5: SciPlex3 Routing-Threshold Sensitivity.

Table S5: SciPlex3 routing-threshold sensitivity.

Threshold	R^2 ↑	Energy Distance ↓	MMD ↓
1.0	0.832	0.363	0.0217
0.9	0.818	0.386	0.0233
**0.8**	**0.891**	**0.234**	**0.0167**
0.7	0.807	0.579	0.0547
0.6	0.800	0.610	0.0662
